# Differentiation between Germinoma and Craniopharyngioma Using Radiomics-Based Machine Learning

**DOI:** 10.3390/jpm12010045

**Published:** 2022-01-04

**Authors:** Boran Chen, Chaoyue Chen, Yang Zhang, Zhouyang Huang, Haoran Wang, Ruoyu Li, Jianguo Xu

**Affiliations:** 1 Department of Neurosurgery, West China Hospital, Sichuan University, Chengdu 610041, China; cbrscu@foxmail.com (B.C.); chaoyuechen01@gmail.com (C.C.); dryangzhang66@gmail.com (Y.Z.); 2 West China School of Medicine, Sichuan University, Chengdu 610041, China; zhouyannhuang@gmail.com (Z.H.); Martywang0917@gmail.com (H.W.); sculiruoyu@gmail.com (R.L.)

**Keywords:** machine learning, radiomics, texture analysis, magnetic resonance imaging, germinoma, craniopharyngioma

## Abstract

For the tumors located in the anterior skull base, germinoma and craniopharyngioma (CP) are unusual types with similar clinical manifestations and imaging features. The difference in treatment strategies and outcomes of patients highlights the importance of making an accurate preoperative diagnosis. This retrospective study enrolled 107 patients diagnosed with germinoma (*n* = 44) and CP (*n* = 63). The region of interest (ROI) was drawn independently by two researchers. Radiomic features were extracted from contrast-enhanced T1WI and T2WI sequences. Here, we established the diagnosis models with a combination of three selection methods, as well as three classifiers. After training the models, their performances were evaluated on the independent validation cohort and compared based on the index of the area under the receiver operating characteristic curve (AUC) in the validation cohort. Nine models were established and compared to find the optimal one defined with the highest AUC in the validation cohort. For the models applied in the contrast-enhanced T1WI images, RF_S_ + RF_C_ and LASSO + LDA were observed to be the optimal models with AUCs of 0.91. For the models applied in the T2WI images, DC + LDA and LASSO + LDA were observed to be the optimal models with AUCs of 0.88. The evidence of this study indicated that radiomics-based machine learning could be potentially considered as the radiological method in the presurgical differential diagnosis of germinoma and CP with a reliable diagnostic performance.

## 1. Introduction

Germ cell tumors (GCTs) mostly occur in pediatric and young adult patients [1]. Germinoma is the most common subtype of GCTs, which accounted for approximately two-thirds of GCTs [2]. The main differential diagnosis of germinoma located in the anterior skull base is craniopharyngioma (CP), an intracranial tumor sharing similar clinical manifestations and imaging features with germinoma. Both of them are located in the suprasellar cistern [3,4], and dominated by non-specific symptoms of an elevated intracranial pressure symptom at the time of diagnosis, such as headache and nausea [4,5,6,7]. Other mutual manifestations include visual impairment, pituitary axis dysfunction, and neurohormonal diabetes insipidus [4,5,6,7]. Alpha-fetoprotein (AFP) and human chorionic gonadotropin (HCG) are suggested as biochemical markers for GCTs [8,9]. While AFP and HCG are not elevated in some GCT cases, histopathologic confirmation is often required for the definitive diagnosis [10]. Furthermore, HCG is sometimes elevated in the serum or cerebrospinal fluid of patients with craniopharyngioma [11,12]. In these cases, AFP and HCG cannot be applied as reliable biomarkers to differentiate between GCT and CP. However, the management of germinoma and CP is quite different. For example, the treatment for localized CP without hypothalamic or optical involvement is recommended as the strategy of total resection [13], while for germinoma, radiotherapy alone or neoadjuvant chemotherapy plus radiotherapy is recommended [14,15].

Magnetic resonance imaging (MRI) is highly suggested in the diagnosis of both tumors for its advantages in excellent soft tissue resolution, multiple plane imaging, non-ionization radiation, and non-iodine contrast agent [16]. However, the image patterns of germinoma and CP were similar to each other, which commonly present as a mixed solid and cystic tumor with contrast enhancement. Considering the differences in treatment strategies and patients’ outcomes, the preoperative diagnosis of these tumors is difficult but crucial, especially for young patients with space-occupying lesions in the sellar or parasellar region [6,7,17].

Texture analysis (TA) is a subset of radiomics technology. The principle of TA is extracting objective and quantitative texture features from images to provide information that can be analyzed with mathematical methods or computer technology [18]. With the ability to extract information that is invisible to the naked eyes, TA has been wildly utilized in medicine to facilitate preoperative diagnosis by MR images and personalized decision-making in the treatment [19]. Previous studies have shown the feasibility of TA-based machine learning models in the radiological diagnosis of various brain tumors [20,21,22]. Therefore, the current study aims to evaluate whether texture features extracted from MR images could be applied in the differentiation between germinoma and CP when combined with machine learning algorithms.

## 2. Materials and Methods

### 2.1. Patient Selection

**Figure 1:**
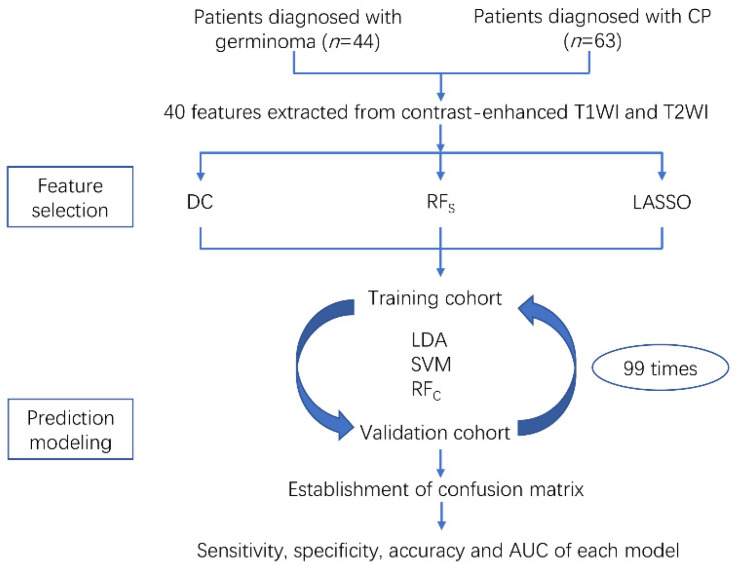
Workflow chart from patient selection to prediction modeling.

Electronic medical records of patients with germinoma or CP in our institution from November 2014 to June 2018 were reviewed. The inclusion criteria of patients were as follows: (1) Pathologic confirmation of germinoma or CP; (2) available high-quality preoperative MR scans performed in the radiological department; (3) the lesion was located in the anterior skull base. The exclusive criteria were as follows: (1) Incomplete medical records in diagnosis or treatment; (2) recorded history of any other intracranial disease; (3) patients had undertaken a treatment, such as surgery, radiotherapy or chemotherapy prior to the available MR scan. The workflow of the current study is shown in Figure 1. This study was approved by the medical ethics committee of West China Hospital (2021-S-851) and the informed consent was waived.

### 2.2. Image Acquisition

Brain MR images of all the patients were examined in the Department of Radiology with the 3.0T GE Scanners before surgery. In the current study, the contrast-enhanced T1-weighted (T1WI) and T2-weighted (T2WI) sequences were chosen to perform TA, since the boundary between the normal brain tissue and tumor is well-circumscribed on these sequences. The parameters of contrast-enhanced T1WI were as follows: TR/TE = 552/10 ms, thickness = 5 mm, FOV = 15 × 15 cm^2^, and data matrix = 256 × 256. Gadopentetate dimeglumine (0.1 mmol/Kg) was the contrast agent for contrast-enhanced images. In addition, the multi-directional data of contrast-enhanced T1WI were collected within 200 s after the injection of gadopentetate dimeglumine. T2WI was acquired before the contrast-enhanced T1WI, and the parameters of T2WI were as follows: TR/TE = 3000/80 ms, thickness = 5 mm, FOV = 19 × 19 cm^2^, and data matrix = 256 × 256.

### 2.3. Radiomic Feature Extraction

**Figure 2:**
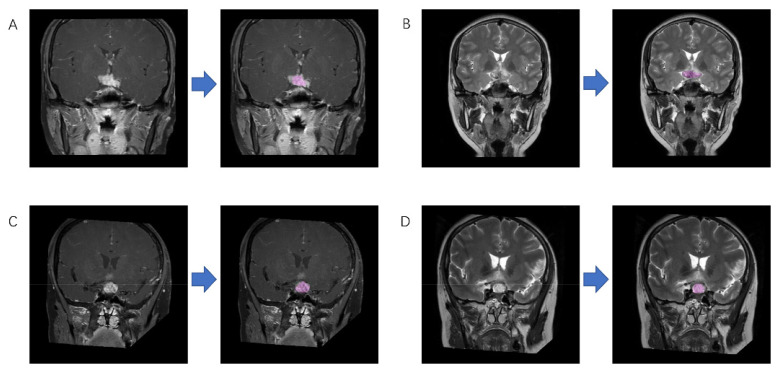
Example of region of interest drawing. This figure shows magnetic resonance imaging of (**A**) germinoma on contrast-enhanced T1WI; (**B**) germinoma on T2WI; (**C**) craniopharyngioma on contrast-enhanced T1WI; (**D**) craniopharyngioma on T2WI before and after drawing.

Two neurosurgeons participated in the extraction of radiomic features using LifeX package (http://www.lifexsoft.org accessed on 6 December 2020) and following the instructions on the website [23]. With the supervision of a senior radiologist with 10 years of experience, the regions of interest (ROI) were drawn along the boundary of the lesions slice-by-slice to obtain three-dimensional radiomic features (Figure 2). Clear cystic components were not included in the ROI since the signal strength of MRI varies with the composition of the cystic contents. Any disagreement on the segmentation was solved by consensus or by the senior radiologist.

In our study, a total of 40 features were extracted from the imaging into the classifier dataset, which were derived from six matrices of two orders. The first-order features, which include the Histogram-based matrix and Shape-based matrix, describe the correlation of voxel intensity distributions. The second-order features, which consist of Gray-level co-occurrence matrix (GLCM), Gray-level run length matrix (GLRLM), neighborhood gray-level dependence matrix (NGLDM), and Gray-level zone length matrix (GLZLM), play a major role in the quantification of radiomic features. The calculation of the first-order features was accomplished through 64 same-size bins and the second-order features were accomplished through grey levels, which were quantized into 64 levels.

### 2.4. Features Selection

In fact, 40 is a relatively large number and some of the features may not be relevant to the differential process. In addition, superabundant features may cause inevitable overfitting. Therefore, we applied three feature-selection methods to select the relevant features, including distance correlation (DC), random forest feature selector (RF_S_), as well as the least absolute shrinkage and selection operator (LASSO). Finally, each feature-selection algorithm generated one feature subset and laid the groundwork for further analysis.

### 2.5. Prediction Modeling

The establishment of the prediction models was based on three classification algorithms, including linear discriminant analysis (LDA, also known as Fisher linear discriminant), support vector machine (SVM), and random forest classifier (RF_C_). With different combinations of selection methods and classifiers, a total of nine models were established, trained, and validated. The dataset was randomly divided into the training cohort and the validation cohort at a ratio of 4 to 1. Feature selection and prediction model training were performed on the training cohort, and then the performances of models were tested on the corresponding validation cohort, which was repeated for 100 cycles. The evaluation of the model performance was based on their diagnostic performance in the validation cohort with the calculation of sensitivity, specificity, accuracy, and the area under the receiver operating characteristic curve (AUC). Here, we used Scikit-learn 0.22, a Python module for machine learning to apply feature selection and classification procedures with the parameters suggested by the developers.

## 3. Results

### 3.1. Patient Characteristics

According to the inclusion and exclusion criteria, we identified 107 patients which consisted of 44 germinomas and 63 CPs. The median age of patients with germinoma was 14 (range 1–44) years, and the age of patients with CP was 30 (range 2–73) years. The male rates of patients with germinoma and CP were 19/44(43.2%) and 37/63(58.7%), respectively. All of the patients had a biopsy of tumor and the diagnoses were made on frozen section pathology, paraffin section pathology, and immunohistochemistry.

### 3.2. Diagnostic Value of Models

In this study, three feature-selection methods and three classifiers were used. In addition, nine diagnostic models were established. Detailed selected radiomic features in each circle are listed in Supplementary Material 1. We sorted the radiomic features by their sum of contribution in the 100 ranking lists in a descending order, and the top six features selected by each feature selector are listed in Table 1.

**Figure 3:**
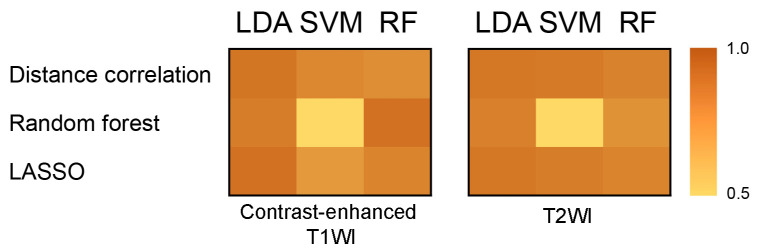
Heatmap of the performances of models based on the area under the receiver operating characteristic curve of the validation cohort, using features extracted from the contrast-enhanced T1WI and T2WI.

The diagnostic values of models were evaluated based on the AUCs in the validation cohort. Regarding the contrast-enhanced T1WI sequence, the RF_S_ + RF_C_ and LASSO + LDA were observed to be the optimal methods with AUCs above 0.9, which were all 0.91 (Table 2, Figure 3), while overfitting was observed in the classifier of SVM when it was combined with RF_S_; see Table 3 regarding the sensitivity, specificity, accuracy, and AUC in the training cohort and validation cohort of RF_S_ + RF_C_ and LASSO + LDA models. A detailed performance of all the models using parameters from the contrast-enhanced T1WI sequence is shown in Supplementary Material 2.

For the T2WI sequence, four patients with germinoma and five patients with CP were not examined before the operation. DC + LDA and LASSO + LDA were observed to be the optimal algorithms with AUCs of 0.88 (Table 2, Figure 3). Overfitting was observed in RF_S_ + SVM again, indicating that this model might be unqualified for the discrimination of germinoma and CP. Table 3 shows the sensitivity, specificity, accuracy, and AUC in the training cohort and validation cohort of DC + LDA and LASSO + LDA models. A detailed performance of all the models using parameters from the T2WI sequence is shown in Supplementary Material 3.

## 4. Discussion

To the best of our knowledge, the current study was the first to apply radiomics-based machine learning in the differentiation between germinoma and CP. Here, we have preliminarily demonstrated that the combination of machine learning algorithms and radiomic features extracted from MR images is helpful in the differential diagnosis of these two types of tumors, providing a new method to assist in conventional radiological diagnosis.

Our results of the combination of TA and machine learning could lead to the development of a novel method that would promote the preoperative diagnosis of germinoma and CP. The accurate preoperative diagnosis of germinoma or CP is crucial in the dramatic differences of the treatment strategies of these two types of tumors. Researches on MRI, the most important examination for intracranial tumors, have shown that some imaging characteristics could be considered significant in the diagnosis. For example, the imaging characteristic of germinoma component is solid, which is predominant with the heterogeneous enhancement of the solid portion on the contrast-enhanced T1WI, while CP is cystic, which is predominant with a marginal enhancement of the multi-cystic lesion [24]. Meanwhile, the apparent diffusion coefficient (ADC) of CP is usually higher than germinoma on diffusion-weighted imaging (DWI) sequences [25]. However, the overall radiological diagnostic accuracy of CP and germinoma was reported to be 87 and 64%, respectively, given the heterogeneity of tumor components as well as the inter- and intra-observer variability [26]. The misdiagnosis could be worse, especially for some germ cell tumor cases with cartilaginous tissue differentiation [27].

Recent researches have applied machine learning technology to the evaluation of neuroimaging in many fields, such as differential diagnosis, biological characterization, treatment response monition, and patient outcome prediction [28,29,30,31]. Radiomic features extracted from MR images are quantitative and the analyzable data are fed into machine learning algorithms. Previously, radiomics-based machine learning studies reported the satisfactory performance of prediction models on the differentiation of primary central nervous system lymphoma and glioblastoma, low-grade glioma and glioblastoma, brain metastasis and glioblastoma, meningioma grading, as well as low- and high-grade gliomas [29,32,33,34,35]. It is expected that radiomics-based machine learning will have a good prospect of application in neuroimaging.

Although the high-throughput TA can provide a large and complex dataset, it makes good use of the whole region of tumor information. However, the dataset usually contains a high level of noise and redundant features. Moreover, it can lead to the high correlation among the extracted features and inevitable risk of overfitting, causing the degeneration of performance. Therefore, the selection of features is necessary. In this study, we applied three selection algorithms, DC, RF_S_, and LASSO, in order to maximize the relevance to the labels of classification. Selection methods are based on the collaboration of feature importance ranking and model estimation. They are divided into three subcategories: “Filter”, in which the score of feature importance does not depend on the given classifier; “wrapper”, which utilizes the classifier of interest to score and rank feature importance; and “embedded”, which embeds features inside the classifier construction, while generating more intricate feature selection and model estimation [36]. “Embedded” and “wrapper” are similar in some aspects. However, “embedded” is more effective as it makes better use of the data and avoids retraining a model from scratch for every feature subset. Among the three selection methods, DC represented “filter”, while RF_S_ and LASSO represented “embedded”. The results showed that the application of different selection algorithms had an impact on the performance of the models. Among the classifiers, LDA represents the linear classifier that classifies two or more classes via a linear combination of features [37]. SVM, a non-linear classifier, constructs a decision hyperplane and achieves the separation of classes by maximizing the margin between the training samples of classes and the hyperplane [37]. RF_C_, a statistically non-parametric classifier, is realized by performing a weighted ensemble of predictive probabilities of de-correlated trees [38,39]. The main advantage of RF is its relatively simple structure, which facilitates the interpretation and visualization of results.

The results of this study showed that the best prediction models were constructed by RF_S_ with RF_C_ and LASSO with LDA in the contrast-enhanced T1WI, as well as DC with LDA and LASSO with LDA in T2WI. Both of the LDA and RF_C_ classifiers had relatively consistent diagnostic performances. While overfitting was observed in the model of RF_S_ + SVM in both of the MRI sequences. We are not able to determine what exactly caused the overfitting, but considering that RF_S_ + RF_C_ achieved the highest AUC of 0.91, we hypothesize that the overfitting was caused by the dependence of SVM on kernel functions and support vectors. We tend to assume that LASSO + LDA can be successfully applied in the presurgical diagnosis of germinoma and CP, due to its robust performance in both contrast-enhanced T1WI and T2WI. However, the variance of diagnostic performance of different selection algorithms might attribute to the relatively small sample size.

There were also several limitations in our study. First, this work was conducted in a single institution. It is unclear whether the results could translate into other institutions or even other patients that were not included in the study since the training and validation processes were performed within a specific population. However, the calculation of radiomic features could be affected by the imaging settings, such as MR scanners and the thickness of slices. Using the radiological data of one center can avoid the inconsistency of imaging settings. Second, the sample size was relatively small. This is a common limitation of other similar studies, which limits the performance of prediction models since it is highly dependent on the training data. Third, this was a retrospective study with an inherent restriction on the inevitable selection bias. Finally, we only extracted radiomic features from two sequences (contrast-enhanced T1WI and T2WI). Features from other sequences, such as fluid-attenuation inversion recovery and DWI, were not evaluated. Further studies are required to assess the diagnostic values of machine learning from other sequences with a larger sample size. Furthermore, the size of ROI was not assessed in this study. However, some features are dependent on the size of ROI, such as SHAPE_Volume (mL), while SHAPE_Volume (mL) was not in the final list of relevant features after summarizing the results of feature selection in the 100 cycles. 

## 5. Conclusions

In conclusion, the evidence of this study indicated that radiomics-based machine learning could facilitate the preoperative differential diagnosis between germinoma and CP. In addition, primary intracranial tumors that have similar clinical manifestations and radiological features but different treatments, had a reliable diagnostic performance. Here, we established high-performance prediction models based on selection methods and classifiers, indicating that this non-invasive approach has the potential to assist in image diagnosis and aid in personalized clinical decision-making.

## Figures and Tables

**Table 1:** The top radiomic features sorted by their sum of contribution in the 100 ranking lists in descending order.

Sequence	Feature Selector	Feature					
Contrast-enhanced T1WI	DC	HISTO_Energy	GLCM_Homogeneity	GLRLM_RP	GLCM_Energy	HISTO_Entropy_log10	GLZLM_ZLNU
	RF_S_	minValue	GLZLM_SZE	GLZLM_LZE	NGLDM_Busyness	GLZLM_LZLGE	GLRLM_HGRE
	LASSO	GLZLM_ZLNU	GLZLM_SZE	HISTO_Energy	HISTO_Entropy_log10	NGLDM_Coarseness	minValue
T2WI	DC	GLRLM_RP	GLRLM_SRE	GLCM_Homogeneity	GLRLM_LRHGE	GLRLM_SRLGE	GLRLM_HGRE
	RF_S_	GLZLM_LZHGE	GLZLM_LZE	GLRLM_HGRE	GLRLM_SRLGE	minValue	GLZLM_SZE
	LASSO	GLCM_Homogeneity	GLZLM_ZLNU	GLRLM_HGRE	NGLDM_Coarseness	GLZLM_SZHGE	GLRLM_RP

Abbreviations: DC: Distance correlation; RF_S_: Random forest feature selector; LASSO: Least absolute shrinkage and selection operator.

**Table 2:** AUCs of the training and validation cohorts in different models using parameters from the contrast-enhanced T1WI or T2WI.

Model	Contrast-Enhanced T1WI		T2WI	
	Training Cohort	Validation Cohort	Training Cohort	Validation Cohort
DC + LDA	0.91	0.89	0.88	0.88
RF_S_ + LDA	0.93	0.86	0.85	0.85
LASSO + LDA	0.97	0.91	0.92	0.88
DC + SVM	0.83	0.82	0.87	0.87
RF_S_ + SVM	1	0.5	1	0.5
LASSO + SVM	0.80	0.75	0.86	0.86
DC + RF_C_	0.89	0.79	0.92	0.84
RF_S_ + RF_C_	0.97	0.91	0.95	0.78
LASSO + RF_C_	0.95	0.83	0.95	0.83

Abbreviations: AUC: Area under the receiver operating characteristic curve; DC: Distance correlation; LDA: Linear discriminant analysis; RF_S_: Random forest feature selector; LASSO: Least absolute shrinkage and selection operator; SVM: Support vector machine; RF_C_: Random forest classifier.

**Table 3:** Diagnostic value of the optimal models using parameters from the contrast-enhanced T1WI or T2WI sequences.

Model	Training Cohort				Validation Cohort			
	Sensitivity	Specificity	Accuracy	AUC	Sensitivity	Specificity	Accuracy	AUC
Contrast-enhanced T1WI								
RF_S_ + RF_C_	0.87	0.95	0.91	0.97	0.81	0.84	0.83	0.91
LASSO + LDA	0.84	0.92	0.89	0.97	0.80	0.84	0.82	0.91
T2WI								
DC + LDA	0.74	0.84	0.80	0.88	0.75	0.81	0.79	0.88
LASSO + LDA	0.75	0.90	0.83	0.92	0.71	0.82	0.77	0.88

Abbreviations: AUC: Area under the receiver operating characteristic curve; RF_S_: Random forest feature selector; RF_C_: Random forest classifier; LASSO: Least absolute shrinkage and selection operator; LDA: Linear discriminant analysis; DC: Distance correlation.

## Data Availability

The datasets generated and analyzed during the current study are available from the corresponding author on reasonable request.

## Supplementary Materials

The following are available online at https://www.mdpi.com/article/10.3390/jpm12010045/s1. Supplementary Material 1: Detailed radiomic features selected by each feature selector in each cycle; Supplementary Material 2: Sensitivity, specificity, accuracy, and the area under the receiver operating characteristic curve of prediction models based on features extracted from contrast-enhanced T1WI; Supplementary Material 3: Sensitivity, specificity, accuracy, and the area under the receiver operating characteristic curve of prediction models based on features extracted from T2WI.

## References

[1] Ostrom Q.T., Cioffi G., Waite K., Kruchko C., Barnholtz-Sloan J.S. (2021). CBTRUS Statistical Report: Primary Brain and Other Central Nervous System Tumors Diagnosed in the United States in 2014–2018. Neuro-Oncology.

[2] Fetcko K., Dey M. (2018). Primary Central Nervous System Germ Cell Tumors: A Review and Update. Med. Res. Arch..

[3] Dufour C., Guerrini-Rousseau L., Grill J. (2014). Central nervous system germ cell tumors: An update. Curr. Opin. Oncol..

[4] Petito C.K., DeGirolami U., Earle K.M. (1976). Craniopharyngiomas: A clinical and pathological review. Cancer.

[5] Jennings M.T., Gelman R., Hochberg F. (1985). Intracranial germ-cell tumors: Natural history and pathogenesis. J. Neurosurg..

[6] Müller H.L., Merchant T.E., Warmuth-Metz M., Martinez-Barbera J.P., Puget S. (2019). Craniopharyngioma. Nat. Rev. Dis. Primers.

[7] Müller H.L. (2014). Craniopharyngioma. Endocr. Rev..

[8] Allen J.C., Nisselbaum J., Epstein F., Rosen G., Schwartz M.K. (1979). Alphafetoprotein and human chorionic gonadotropin determination in cerebrospinal fluid. An aid to the diagnosis and management of intracranial germ-cell tumors. J. Neurosurg..

[9] Haase J., Borgaard-Pedersen B. (1979). Alpha-feto-protein (AFP) and human chorionic gonadotropin (HCG) as biochemical markers of intracranial germ-cell tumours. Acta Neurochir..

[10] Qaddoumi I., Sane M., Li S., Kocak M., Pai-Panandiker A., Harreld J., Klimo P., Wright K., Broniscer A., Gajjar A. (2012). Diagnostic utility and correlation of tumor markers in the serum and cerebrospinal fluid of children with intracranial germ cell tumors. Childs Nerv. Syst. ChNS Off. J. Int. Soc. Pediatric Neurosurg..

[11] Honegger J., Mann K., Thierauf P., Zrinzo A., Fahlbusch R. (1995). Human chorionic gonadotrophin immunoactivity in cystic intracranial tumours. Clin. Endocrinol..

[12] Kinoshita Y., Tominaga A., Usui S., Kurisu K. (2014). A craniopharyngioma with spontaneous involution of a gadolinium-enhanced region on magnetic resonance imaging. Surg. Neurol. Int..

[13] Buchfelder M., Schlaffer S.M., Lin F., Kleindienst A. (2013). Surgery for craniopharyngioma. Pituitary.

[14] Claude L., Faure-Conter C., Frappaz D., Mottolese C., Carrie C. (2015). Radiation therapy in pediatric pineal tumors. Neuro-Chirurgie.

[15] Frappaz D., Conter C.F., Szathmari A., Valsijevic A., Mottolese C. (2015). The management of pineal tumors as a model for a multidisciplinary approach in neuro-oncology. Neuro-Chirurgie.

[16] Fisher A.R., Siegelman E.S. (2002). Magnetic resonance imaging techniques. Clin. Liver Dis..

[17] Fujisawa I., Asato R., Okumura R., Nakano Y., Shibata T., Hamanaka D., Hashimoto T., Konishi J. (1991). Magnetic resonance imaging of neurohypophyseal germinomas. Cancer.

[18] Gillies R.J., Kinahan P.E., Hricak H. (2016). Radiomics: Images Are More than Pictures, They Are Data. Radiology.

[19] Deo R.C. (2015). Machine Learning in Medicine. Circulation.

[20] Amin J., Sharif M., Raza M., Saba T., Anjum M.A. (2019). Brain tumor detection using statistical and machine learning method. Comput. Methods Programs Biomed..

[21] Artzi M., Bressler I., Ben Bashat D. (2019). Differentiation between glioblastoma, brain metastasis and subtypes using radiomics analysis. J. Magn. Reson. Imaging JMRI.

[22] Kniep H.C., Madesta F., Schneider T., Hanning U., Schonfeld M.H., Schon G., Fiehler J., Gauer T., Werner R., Gellissen S. (2019). Radiomics of Brain MRI: Utility in Prediction of Metastatic Tumor Type. Radiology.

[23] Nioche C., Orlhac F., Boughdad S., Reuze S., Goya-Outi J., Robert C., Pellot-Barakat C., Soussan M., Frouin F., Buvat I. (2018). LIFEx: A Freeware for Radiomic Feature Calculation in Multimodality Imaging to Accelerate Advances in the Characterization of Tumor Heterogeneity. Cancer Res..

[24] Lee H.J., Wu C.C., Wu H.M., Hung S.C., Lirng J.F., Luo C.B., Chang F.C., Guo W.Y. (2015). Pretreatment diagnosis of suprasellar papillary craniopharyngioma and germ cell tumors of adult patients. AJNR Am. J. Neuroradiol..

[25] Kinoshita Y., Yamasaki F., Tominaga A., Ohtaki M., Usui S., Arita K., Sugiyama K., Kurisu K. (2016). Diffusion-weighted imaging and the apparent diffusion coefficient on 3T MR imaging in the differentiation of craniopharyngiomas and germ cell tumors. Neurosurg. Rev..

[26] Norris G.A., Garcia J., Hankinson T.C., Handler M., Foreman N., Mirsky D., Stence N., Dorris K., Green A.L. (2019). Diagnostic accuracy of neuroimaging in pediatric optic chiasm/sellar/suprasellar tumors. Pediatric Blood Cancer.

[27] Chang C.V., Nunes Vdos S., Felicio A.C., Zanini M.A., Cunha-Neto M.B., de Castro A.V. (2008). Mixed germ cell tumor of the pituitary-hypothalamic region presenting as craniopharyngioma: Case report and review of the literature. Arq. Bras. Endocrinol. Metabol..

[28] Grossmann P., Narayan V., Chang K., Rahman R., Abrey L., Reardon D.A., Schwartz L.H., Wen P.Y., Alexander B.M., Huang R. (2017). Quantitative imaging biomarkers for risk stratification of patients with recurrent glioblastoma treated with bevacizumab. Neuro-Oncology.

[29] Kang D., Park J.E., Kim Y.H., Kim J.H., Oh J.Y., Kim J., Kim Y., Kim S.T., Kim H.S. (2018). Diffusion radiomics as a diagnostic model for atypical manifestation of primary central nervous system lymphoma: Development and multicenter external validation. Neuro-Oncology.

[30] Larroza A., Moratal D., Paredes-Sanchez A., Soria-Olivas E., Chust M.L., Arribas L.A., Arana E. (2015). Support vector machine classification of brain metastasis and radiation necrosis based on texture analysis in MRI. J. Magn. Reson. Imaging JMRI.

[31] Zhou H., Vallieres M., Bai H.X., Su C., Tang H., Oldridge D., Zhang Z., Xiao B., Liao W., Tao Y. (2017). MRI features predict survival and molecular markers in diffuse lower-grade gliomas. Neuro-Oncology.

[32] Cao H., Erson-Omay E.Z., Li X., Gunel M., Moliterno J., Fulbright R.K. (2020). A quantitative model based on clinically relevant MRI features differentiates lower grade gliomas and glioblastoma. Eur. Radiol..

[33] Park Y.W., Oh J., You S.C., Han K., Ahn S.S., Choi Y.S., Chang J.H., Kim S.H., Lee S.K. (2019). Radiomics and machine learning may accurately predict the grade and histological subtype in meningiomas using conventional and diffusion tensor imaging. Eur. Radiol..

[34] Tateishi M., Nakaura T., Kitajima M., Uetani H., Nakagawa M., Inoue T., Kuroda J.I., Mukasa A., Yamashita Y. (2020). An initial experience of machine learning based on multi-sequence texture parameters in magnetic resonance imaging to differentiate glioblastoma from brain metastases. J. Neurol. Sci..

[35] Zhou H., Chang K., Bai H.X., Xiao B., Su C., Bi W.L., Zhang P.J., Senders J.T., Vallières M., Kavouridis V.K. (2019). Machine learning reveals multimodal MRI patterns predictive of isocitrate dehydrogenase and 1p/19q status in diffuse low- and high-grade gliomas. J. Neuro-Oncol..

[36] Guyon I., Elisseeff A. (2003). An Introduction to Variable and Feature Selection. J. Mach. Learn. Res..

[37] Dellacasa Bellingegni A., Gruppioni E., Colazzo G., Davalli A., Sacchetti R., Guglielmelli E., Zollo L. (2017). NLR, MLP, SVM, and LDA: A comparative analysis on EMG data from people with trans-radial amputation. J. Neuroeng. Rehabil..

[38] Azar A.T., Elshazly H.I., Hassanien A.E., Elkorany A.M. (2014). A random forest classifier for lymph diseases. Comput. Methods Programs Biomed..

[39] Quadrianto N., Ghahramani Z. (2015). A Very Simple Safe-Bayesian Random Forest. IEEE Trans. Pattern Anal. Mach. Intell..

