# Supplementary material for: Differentiation between Germinoma and Craniopharyngioma Using Radiomics-Based Machine Learning
Source: J Pers Med. 2022 Jan 4;12(1):45. doi: 10.3390/jpm12010045 (PMC8778008; doi:10.3390/jpm12010045)
Supplement: Supplementary file 1 [file jpm-12-00045-s001.zip › jpm-1511549-supplementary/Supplementary Material 1.pdf]

Contrast-enhanced T1WI. Feature selector: DC

Cicle	Feature					
1	HISTO_Energy	GLCM_Homogeneit	GLRLM_RP	HISTO_Entropy_log	GLRLM_SRE	GLCM_Energy
2	HISTO_Energy	GLCM_Energy	GLRLM_RP	HISTO_Entropy_log	GLCM_Homogeneit	GLRLM_SRE
3	HISTO_Energy	HISTO_Entropy_log	GLCM_Homogeneit	GLRLM_RP	GLCM_Energy	GLRLM_SRE
4	GLRLM_RP	GLCM_Homogeneit	HISTO_Energy	GLCM_Energy	HISTO_Entropy_log	GLRLM_SRE
5	HISTO_Energy	HISTO_Entropy_log	GLZLM_ZLNU	SHAPE_Volume (ml	GLRLM_RP	GLCM_Homogeneity
6	HISTO_Energy	HISTO_Entropy_log	GLCM_Homogeneit	GLCM_Energy	GLZLM_ZLNU	SHAPE_Volume (mL
7	SHAPE_Volume (ml	GLZLM_ZLNU	HISTO_Energy	GLCM_Homogeneit	GLRLM_RP	GLCM_Energy
8	GLCM_Homogeneit	HISTO_Energy	HISTO_Entropy_log	GLRLM_RP	GLZLM_ZLNU	GLCM_Energy
9	HISTO_Energy	HISTO_Entropy_log	GLCM_Homogeneit	GLRLM_RP	GLCM_Energy	GLRLM_SRE
10	GLZLM_ZLNU	HISTO_Energy	HISTO_Entropy_log	GLCM_Energy	GLCM_Homogeneit	GLRLM_RP
11	HISTO_Energy	GLCM_Homogeneit	GLRLM_RP	GLCM_Energy	HISTO_Entropy_log	GLRLM_SRE
12	HISTO_Energy	GLRLM_RP	GLZLM_ZLNU	GLCM_Energy	GLCM_Homogeneit	HISTO_Entropy_log1
13	GLCM_Homogeneit	HISTO_Energy	HISTO_Entropy_log	GLRLM_RP	GLZLM_ZLNU	GLCM_Energy
14	HISTO_Energy	HISTO_Entropy_log	GLRLM_RP	GLCM_Homogeneit	GLCM_Energy	GLZLM_ZLNU
15	HISTO_Energy	GLRLM_RP	GLCM_Homogeneit	GLCM_Energy	SHAPE_Volume (ml	HISTO_Entropy_log1
16	HISTO_Energy	HISTO_Entropy_log	GLCM_Energy	GLCM_Homogeneit	GLRLM_LRHGE	GLZLM_HGZE
17	HISTO_Energy	GLZLM_ZLNU	GLCM_Energy	HISTO_Entropy_log	GLCM_Homogeneit	GLRLM_RP
18	HISTO_Energy	HISTO_Entropy_log	GLCM_Homogeneit	GLRLM_RP	GLCM_Energy	GLRLM_SRHGE
19	HISTO_Energy	GLCM_Homogeneit	GLRLM_RP	HISTO_Entropy_log	GLCM_Energy	GLRLM_SRE
20	GLZLM_ZLNU	GLRLM_RP	HISTO_Energy	GLCM_Homogeneit	NGLDM_Coarsenes	GLCM_Energy
21	HISTO_Energy	GLRLM_RP	GLCM_Homogeneit	GLCM_Energy	GLRLM_SRE	HISTO_Entropy_log1
22	GLRLM_RP	GLCM_Homogeneit	HISTO_Entropy_log	HISTO_Energy	GLRLM_SRE	GLZLM_ZLNU
23	GLRLM_RP	GLCM_Homogeneit	GLRLM_SRE	HISTO_Energy	HISTO_Entropy_log	GLCM_Energy
24	GLZLM_ZLNU	SHAPE_Volume (ml	GLCM_Homogeneit	HISTO_Energy	GLRLM_RP	HISTO_Entropy_log1
25	HISTO_Energy	GLZLM_ZLNU	HISTO_Entropy_log	GLCM_Energy	GLRLM_RP	GLCM_Homogeneity
26	GLZLM_ZLNU	HISTO_Energy	GLCM_Energy	GLCM_Homogeneit	GLRLM_RP	HISTO_Entropy_log1
27	HISTO_Energy	HISTO_Entropy_log	GLCM_Energy	GLCM_Homogeneit	GLRLM_RP	GLZLM_ZLNU
28	GLCM_Homogeneit	HISTO_Energy	GLRLM_RP	HISTO_Entropy_log	GLRLM_SRE	GLCM_Energy
29	HISTO_Energy	HISTO_Entropy_log	GLCM_Energy	GLCM_Homogeneit	GLRLM_RP	GLZLM_ZLNU
30	GLCM_Energy	HISTO_Energy	GLZLM_ZLNU	GLCM_Homogeneit	GLRLM_RP	HISTO_Entropy_log1
31	HISTO_Energy	HISTO_Entropy_log	GLCM_Energy	GLCM_Homogeneit	GLRLM_RP	GLRLM_SRE
32	HISTO_Energy	GLCM_Homogeneit	HISTO_Entropy_log	GLRLM_RP	GLRLM_SRE	GLCM_Energy
33	GLRLM_RP	GLCM_Homogeneit	NGLDM_Busyness	SHAPE_Volume (ml	GLRLM_SRE	HISTO_Energy
34	HISTO_Energy	SHAPE_Volume (ml	GLZLM_ZLNU	GLCM_Energy	GLRLM_RP	GLCM_Homogeneity
35	GLCM_Homogeneit	HISTO_Energy	HISTO_Entropy_log	GLRLM_RP	GLCM_Energy	GLZLM_HGZE
36	GLZLM_ZLNU	GLCM_Energy	NGLDM_Busyness	HISTO_Energy	GLRLM_RP	minValue
37	GLRLM_RP	GLCM_Homogeneit	GLRLM_SRE	HISTO_Energy	minValue	HISTO_Entropy_log1
38	GLZLM_ZLNU	HISTO_Energy	GLCM_Energy	GLRLM_RP	NGLDM_Busyness	GLCM_Homogeneity
39	GLZLM_ZLNU	minValue	HISTO_Energy	GLRLM_RP	GLCM_Energy	GLCM_Homogeneity
40	GLZLM_ZLNU	SHAPE_Volume (ml	NGLDM_Busyness	HISTO_Energy	GLCM_Homogeneit	GLRLM_RP
41	GLRLM_RP	GLCM_Homogeneit	GLRLM_SRE	HISTO_Energy	GLCM_Energy	HISTO_Entropy_log1
42	HISTO_Energy	GLCM_Energy	GLRLM_RP	GLRLM_SRE	HISTO_Entropy_log	GLCM_Homogeneity
43	GLCM_Homogeneit	GLRLM_RP	GLZLM_ZLNU	HISTO_Entropy_log	HISTO_Energy	GLRLM_SRE
44	GLCM_Homogeneit	GLRLM_RP	HISTO_Energy	minValue	GLCM_Energy	HISTO_Entropy_log1
45	HISTO_Energy	HISTO_Entropy_log	GLCM_Homogeneit	GLCM_Energy	GLRLM_RP	GLZLM_ZLNU
46	HISTO_Energy	GLCM_Energy	GLCM_Homogeneit	GLRLM_RP	HISTO_Entropy_log	GLRLM_SRE
47	HISTO_Energy	HISTO_Entropy_log	SHAPE_Volume (ml	GLCM_Energy	GLZLM_ZLNU	GLCM_Homogeneity
48	HISTO_Energy	GLCM_Energy	GLCM_Homogeneit	HISTO_Entropy_log	minValue	GLRLM_RP
49	HISTO_Energy	HISTO_Entropy_log	GLCM_Homogeneit	GLRLM_RP	GLCM_Energy	GLRLM_SRE
50	HISTO_Energy	GLCM_Energy	SHAPE_Volume (ml	HISTO_Entropy_log	GLZLM_ZLNU	GLCM_Homogeneity
51	GLCM_Energy	HISTO_Energy	GLCM_Homogeneit	GLRLM_RP	HISTO_Entropy_log	SHAPE_Volume (mL
52	SHAPE_Volume (ml	GLCM_Homogeneit	HISTO_Energy	GLRLM_RP	GLZLM_ZLNU	GLCM_Energy
53	GLRLM_RP	HISTO_Energy	GLCM_Homogeneit	GLCM_Energy	GLRLM_SRE	HISTO_Entropy_log1
54	HISTO_Energy	GLCM_Homogeneit	HISTO_Entropy_log	GLZLM_ZLNU	GLCM_Energy	GLRLM_RP
55	HISTO_Energy	GLRLM_RP	GLCM_Homogeneit	NGLDM_Busyness	HISTO_Entropy_log	GLRLM_SRE
56	HISTO_Energy	GLCM_Homogeneit	HISTO_Entropy_log	GLRLM_RP	GLCM_Energy	GLZLM_ZLNU
57	HISTO_Energy	HISTO_Entropy_log	GLCM_Homogeneit	GLCM_Energy	GLRLM_RP	GLRLM_SRE
58	HISTO_Energy	HISTO_Entropy_log	GLCM_Homogeneit	GLZLM_ZLNU	GLRLM_RP	NGLDM_Coarseness
59	GLZLM_ZLNU	GLRLM_RP	GLCM_Homogeneit	HISTO_Energy	SHAPE_Volume (ml	NGLDM_Coarseness
60	GLZLM_ZLNU	NGLDM_Coarsenes	SHAPE_Volume (ml	GLCM_Homogeneit	HISTO_Energy	GLRLM_RP
61	GLZLM_ZLNU	HISTO_Energy	GLCM_Energy	GLCM_Homogeneit	HISTO_Entropy_log	SHAPE_Volume (mL
62	HISTO_Energy	GLCM_Energy	HISTO_Entropy_log	GLCM_Homogeneit	GLRLM_RP	GLZLM_ZLNU
63	HISTO_Energy	GLZLM_ZLNU	HISTO_Entropy_log	minValue	GLCM_Energy	GLCM_Homogeneity
64	HISTO_Energy	GLCM_Homogeneit	GLRLM_RP	HISTO_Entropy_log	GLRLM_SRE	GLCM_Energy
65	HISTO_Energy	HISTO_Entropy_log	GLCM_Homogeneit	GLCM_Energy	GLRLM_RP	GLRLM_SRE
66	HISTO_Energy	GLCM_Energy	GLZLM_ZLNU	HISTO_Entropy_log	GLCM_Homogeneit	GLRLM_RP
67	HISTO_Energy	GLZLM_ZLNU	GLCM_Energy	GLCM_Homogeneit	HISTO_Entropy_log	GLRLM_RP
68	HISTO_Energy	GLCM_Energy	HISTO_Entropy_log	GLCM_Homogeneit	GLRLM_RP	GLCM_Entropy_log1
69	GLCM_Energy	HISTO_Energy	GLZLM_ZLNU	GLRLM_RP	GLCM_Homogeneit	NGLDM_Coarseness
70	GLRLM_RP	GLCM_Homogeneit	HISTO_Energy	GLRLM_SRE	HISTO_Entropy_log	GLCM_Energy
71	HISTO_Energy	GLCM_Energy	GLRLM_RP	HISTO_Entropy_log	GLCM_Homogeneit	GLRLM_SRE
72	HISTO_Energy	GLCM_Energy	GLZLM_ZLNU	HISTO_Entropy_log	SHAPE_Volume (ml	GLCM_Homogeneity
73	HISTO_Energy	GLCM_Energy	HISTO_Entropy_log	GLZLM_ZLNU	GLZLM_SZE	GLCM_Homogeneity
74	GLZLM_ZLNU	GLCM_Energy	GLRLM_RP	NGLDM_Busyness	HISTO_Energy	GLZLM_GLNU

75	GLCM_Homogeneit	GLRLM_RP	HISTO_Energy	HISTO_Entropy_log	NGLDM_Coarsenes	GLRLM_SRE
76	HISTO_Energy	GLCM_Homogeneit	HISTO_Entropy_log	GLRLM_SRHGE	GLRLM_RP	GLCM_Energy
77	SHAPE_Volume (ml	HISTO_Energy	GLZLM_ZLNU	NGLDM_Busyness	GLCM_Energy	GLRLM_SRHGE
78	HISTO_Energy	GLCM_Energy	HISTO_Entropy_log	GLCM_Homogeneit	GLRLM_RP	GLZLM_ZLNU
79	HISTO_Energy	GLRLM_RP	GLCM_Energy	GLCM_Homogeneit	GLRLM_SRE	HISTO_Entropy_log
80	HISTO_Energy	GLRLM_RP	GLCM_Energy	GLCM_Homogeneit	GLRLM_SRE	HISTO_Entropy_log
81	GLRLM_RP	GLCM_Homogeneit	HISTO_Energy	HISTO_Entropy_log	GLRLM_SRE	NGLDM_Coarsenes
82	GLCM_Homogeneit	HISTO_Entropy_log	HISTO_Energy	GLRLM_RP	NGLDM_Coarsenes	GLRLM_SRHGE
83	SHAPE_Volume (ml	GLCM_Homogeneit	GLRLM_RP	GLZLM_ZLNU	HISTO_Energy	GLRLM_SRE
84	HISTO_Energy	GLCM_Homogeneit	HISTO_Entropy_log	GLRLM_RP	GLCM_Energy	GLRLM_SRE
85	HISTO_Energy	GLZLM_ZLNU	HISTO_Entropy_log	GLCM_Energy	SHAPE_Volume (ml	GLCM_Homogeneity
86	GLCM_Energy	HISTO_Energy	GLZLM_ZLNU	GLRLM_RP	HISTO_Entropy_log	SHAPE_Volume (mL
87	HISTO_Energy	SHAPE_Volume (ml	GLZLM_ZLNU	NGLDM_Busyness	GLCM_Homogeneit	HISTO_Entropy_log
88	GLCM_Homogeneit	GLRLM_RP	NGLDM_Coarsenes	GLRLM_SRE	GLZLM_ZLNU	GLRLM_GLNU
89	GLRLM_RP	GLCM_Homogeneit	HISTO_Energy	GLRLM_SRE	GLCM_Energy	HISTO_Entropy_log
90	SHAPE_Volume (ml	minValue	GLZLM_ZLNU	GLCM_Energy	HISTO_Energy	GLRLM_RP
91	HISTO_Energy	GLCM_Energy	SHAPE_Volume (ml	GLZLM_ZLNU	GLCM_Homogeneit	HISTO_Entropy_log
92	HISTO_Energy	GLCM_Energy	HISTO_Entropy_log	GLCM_Homogeneit	GLRLM_RP	GLZLM_ZLNU
93	HISTO_Energy	GLCM_Homogeneit	HISTO_Entropy_log	GLCM_Energy	GLRLM_RP	GLRLM_SRHGE
94	HISTO_Energy	HISTO_Entropy_log	GLCM_Energy	GLCM_Homogeneit	GLRLM_RP	GLZLM_ZLNU
95	HISTO_Energy	GLCM_Energy	HISTO_Entropy_log	GLCM_Homogeneit	GLRLM_RP	SHAPE_Volume (mL
96	HISTO_Energy	GLCM_Homogeneit	HISTO_Entropy_log	GLRLM_RP	GLCM_Energy	GLRLM_SRE
97	GLCM_Homogeneit	GLRLM_RP	HISTO_Energy	HISTO_Entropy_log	GLRLM_SRE	GLCM_Energy
98	GLCM_Homogeneit	GLRLM_RP	GLCM_Energy	HISTO_Energy	GLRLM_SRE	HISTO_Entropy_log
99	GLZLM_ZLNU	NGLDM_Coarsenes	HISTO_Energy	GLRLM_RP	SHAPE_Volume (ml	GLCM_Homogeneity
100	HISTO_Energy	GLCM_Homogeneit	HISTO_Entropy_log	GLRLM_RP	GLZLM_ZLNU	minValue

Contrast-enhanced T1WI. Feature selector: RFs

Cicle	Feature					
1	minValue	GLRLM_LRE	HISTO_Energy	GLZLM_SZE	GLZLM_LZLGE	GLZLM_LZE
2	minValue	GLZLM_LZE	HISTO_Skewness	HISTO_Energy	SHAPE_Volume (mL	GLZLM_LZLGE
3	NGLDM_Busyness	GLZLM_SZE	GLZLM_LZE	minValue	SHAPE_Volume (mL	GLZLM_ZP
4	minValue	GLZLM_SZE	NGLDM_Busyness	GLZLM_LZE	GLZLM_ZLNU	GLZLM_HGZE
5	NGLDM_Busyness	GLZLM_SZE	GLZLM_LZE	minValue	GLZLM_ZLNU	GLRLM_GLNU
6	minValue	GLZLM_SZE	HISTO_Energy	GLRLM_HGRE	stdValue	GLZLM_ZLNU
7	minValue	NGLDM_Busyness	GLZLM_SZE	GLRLM_LRHGE	GLZLM_ZLNU	GLRLM_HGRE
8	minValue	GLZLM_SZE	GLZLM_LZHGE	GLZLM_LZE	GLCM_Homogeneity	HISTO_Energy
9	minValue	GLZLM_SZE	GLZLM_LZE	GLZLM_LZHGE	HISTO_Energy	GLCM_Homogeneity
10	GLZLM_SZE	minValue	GLZLM_LZE	GLRLM_HGRE	stdValue	GLRLM_LRHGE
11	minValue	GLZLM_LZE	GLZLM_SZE	GLZLM_LZLGE	GLRLM_HGRE	NGLDM_Coarseness
12	minValue	GLZLM_SZE	GLZLM_LZE	SHAPE_Volume (mL	NGLDM_Busyness	GLZLM_LZHGE
13	GLZLM_SZE	minValue	GLZLM_ZLNU	GLZLM_LZE	GLZLM_LZLGE	HISTO_Energy
14	minValue	GLZLM_LZE	GLZLM_SZE	GLZLM_ZLNU	GLRLM_HGRE	NGLDM_Coarseness
15	GLZLM_SZE	NGLDM_Busyness	minValue	GLZLM_LZE	GLZLM_HGZE	GLZLM_SZLGE
16	minValue	GLZLM_SZE	GLZLM_LZE	GLZLM_HGZE	GLZLM_LZLGE	GLRLM_LRE
17	minValue	GLZLM_LZE	GLZLM_SZE	NGLDM_Busyness	GLZLM_ZLNU	HISTO_Energy
18	GLZLM_SZE	minValue	GLRLM_HGRE	GLZLM_LZE	GLZLM_LZLGE	GLRLM_LRHGE
19	minValue	HISTO_Energy	GLZLM_SZE	GLZLM_LZE	GLRLM_GLNU	GLZLM_LZHGE
20	GLZLM_SZE	minValue	GLZLM_LZLGE	GLZLM_LZE	NGLDM_Busyness	GLZLM_ZLNU
21	minValue	GLZLM_LZE	GLZLM_LZHGE	stdValue	GLZLM_SZE	HISTO_Energy
22	GLZLM_LZE	minValue	GLZLM_SZE	NGLDM_Busyness	GLZLM_LZLGE	GLZLM_LZHGE
23	GLZLM_LZE	GLZLM_LZLGE	GLCM_Homogeneity	minValue	GLRLM_LRE	GLRLM_HGRE
24	GLZLM_SZE	minValue	NGLDM_Busyness	GLZLM_LZLGE	SHAPE_Volume (mL	GLZLM_ZLNU
25	minValue	GLZLM_SZE	GLZLM_ZLNU	NGLDM_Busyness	HISTO_Energy	GLZLM_LZLGE
26	minValue	GLZLM_SZE	GLCM_Homogeneity	GLZLM_ZLNU	GLZLM_LZE	GLCM_Energy
27	minValue	HISTO_Energy	GLZLM_LZLGE	GLZLM_LZE	GLZLM_ZP	stdValue
28	GLZLM_SZE	NGLDM_Busyness	minValue	GLZLM_LZE	GLZLM_LZHGE	GLZLM_LZLGE
29	GLZLM_SZE	GLRLM_HGRE	HISTO_Energy	minValue	GLZLM_ZLNU	NGLDM_Busyness
30	GLZLM_SZE	minValue	GLZLM_LZE	GLZLM_LZLGE	GLZLM_SZLGE	SHAPE_Volume (mL)
31	minValue	GLZLM_SZE	GLCM_Homogeneity	GLZLM_LZE	GLRLM_HGRE	HISTO_Energy
32	minValue	GLZLM_SZE	GLZLM_LZE	NGLDM_Busyness	SHAPE_Volume (mL	NGLDM_Coarseness
33	GLZLM_LZE	NGLDM_Busyness	GLZLM_SZE	minValue	GLCM_Homogeneity	GLZLM_LZLGE
34	minValue	GLZLM_SZE	SHAPE_Volume (mL	GLZLM_LZLGE	GLZLM_LZE	NGLDM_Busyness
35	GLZLM_LZE	minValue	GLZLM_SZE	GLCM_Homogeneity	GLRLM_HGRE	GLZLM_LZLGE
36	minValue	GLZLM_SZE	NGLDM_Busyness	GLZLM_LZE	GLZLM_ZLNU	GLZLM_LZLGE
37	minValue	GLZLM_SZE	NGLDM_Busyness	GLZLM_LZE	GLZLM_LZLGE	GLZLM_LZHGE
38	GLZLM_SZE	GLZLM_LZE	minValue	NGLDM_Busyness	GLZLM_ZLNU	GLZLM_LZLGE
39	minValue	GLZLM_SZE	NGLDM_Busyness	GLZLM_LZE	GLZLM_LZLGE	GLZLM_ZLNU
40	minValue	GLZLM_SZE	GLZLM_LZE	SHAPE_Volume (mL	NGLDM_Busyness	GLRLM_HGRE
41	GLZLM_LZE	minValue	HISTO_Energy	NGLDM_Busyness	GLZLM_LZLGE	GLRLM_HGRE
42	GLZLM_SZE	minValue	GLZLM_LZE	NGLDM_Busyness	HISTO_Energy	GLRLM_HGRE
43	GLZLM_LZE	minValue	GLZLM_LZHGE	HISTO_Energy	GLRLM_HGRE	NGLDM_Busyness
44	minValue	GLZLM_SZE	GLZLM_LZE	GLZLM_LZLGE	GLZLM_LZHGE	GLCM_Homogeneity
45	GLRLM_HGRE	GLZLM_SZE	GLZLM_LZE	minValue	NGLDM_Busyness	HISTO_Energy
46	minValue	GLZLM_SZE	GLZLM_LZE	NGLDM_Busyness	HISTO_Energy	GLZLM_LZLGE

47	minValue	GLZLM_SZE	NGLDM_Busyness	GLRLM_HGRE	GLRLM_LRHGE	HISTO_Energy
48	GLZLM_SZE	minValue	GLZLM_LZE	GLZLM_LZLGE	GLRLM_HGRE	NGLDM_Busyness
49	minValue	GLZLM_SZE	GLRLM_HGRE	GLCM_Homogeneity	GLZLM_LZLGE	SHAPE_Volume (mL)
50	minValue	GLZLM_SZE	SHAPE_Volume (mL	GLRLM_LRHGE	GLZLM_LZE	HISTO_Energy
51	minValue	GLZLM_SZE	GLZLM_LZE	SHAPE_Volume (mL	GLZLM_LZLGE	NGLDM_Busyness
52	minValue	GLZLM_LZE	SHAPE_Volume (mL	NGLDM_Busyness	GLRLM_HGRE	stdValue
53	minValue	GLZLM_LZLGE	GLZLM_SZE	GLRLM_LRE	GLZLM_LZHGE	GLZLM_LZE
54	minValue	GLZLM_SZE	GLZLM_LZLGE	GLZLM_LZE	GLCM_Homogeneity	stdValue
55	minValue	GLRLM_LRE	GLCM_Homogeneity	GLZLM_LZHGE	stdValue	GLZLM_SZE
56	GLZLM_SZE	minValue	GLZLM_ZLNU	GLCM_Homogeneity	NGLDM_Busyness	GLRLM_LRLGE
57	minValue	GLZLM_SZE	GLZLM_LZE	GLRLM_HGRE	NGLDM_Busyness	stdValue
58	minValue	GLZLM_SZE	GLZLM_LZLGE	GLZLM_LZE	HISTO_Energy	GLZLM_ZP
59	minValue	NGLDM_Busyness	GLZLM_LZE	GLZLM_SZE	GLRLM_HGRE	stdValue
60	GLZLM_SZE	GLZLM_LZE	NGLDM_Busyness	minValue	GLZLM_ZLNU	GLZLM_LZLGE
61	GLZLM_SZE	NGLDM_Busyness	minValue	GLCM_Homogeneity	GLZLM_LZE	GLZLM_SZLGE
62	minValue	GLZLM_SZE	GLZLM_ZLNU	GLZLM_LZLGE	SHAPE_Volume (mL	GLRLM_HGRE
63	GLZLM_SZE	minValue	GLZLM_LZE	GLZLM_LZLGE	NGLDM_Busyness	GLZLM_ZLNU
64	minValue	GLZLM_LZE	NGLDM_Busyness	GLZLM_SZE	GLZLM_LZLGE	NGLDM_Coarseness
65	minValue	GLZLM_LZLGE	GLZLM_SZE	HISTO_Energy	GLZLM_LZE	GLRLM_HGRE
66	GLZLM_LZE	minValue	GLZLM_SZE	NGLDM_Busyness	GLZLM_LZLGE	HISTO_Energy
67	GLZLM_SZE	minValue	NGLDM_Busyness	GLZLM_LZE	GLZLM_ZLNU	SHAPE_Volume (mL)
68	GLZLM_SZE	minValue	GLZLM_LZE	HISTO_Energy	stdValue	GLRLM_HGRE
69	GLZLM_SZE	minValue	GLZLM_LZE	GLCM_Homogeneity	GLZLM_LZLGE	stdValue
70	minValue	GLZLM_SZE	GLZLM_LZE	NGLDM_Coarseness	NGLDM_Busyness	GLZLM_LZLGE
71	minValue	GLZLM_LZLGE	GLRLM_HGRE	NGLDM_Busyness	GLCM_Homogeneity	GLZLM_HGZE
72	minValue	GLZLM_SZE	GLZLM_LZE	stdValue	GLZLM_GLNU	maxValue
73	GLZLM_SZE	NGLDM_Busyness	GLZLM_LZE	GLRLM_LRHGE	GLZLM_HGZE	GLRLM_GLNU
74	GLZLM_SZE	minValue	GLZLM_LZE	SHAPE_Volume (mL	NGLDM_Busyness	GLRLM_LRHGE
75	GLZLM_LZE	NGLDM_Busyness	minValue	HISTO_Energy	GLZLM_SZE	GLRLM_LRE
76	minValue	GLZLM_LZLGE	GLZLM_LZE	GLRLM_HGRE	GLCM_Homogeneity	HISTO_Energy
77	minValue	GLZLM_SZE	GLZLM_ZLNU	NGLDM_Busyness	SHAPE_Volume (mL	maxValue
78	GLZLM_LZLGE	minValue	NGLDM_Busyness	GLZLM_SZE	stdValue	SHAPE_Volume (mL)
79	minValue	GLZLM_SZE	GLZLM_LZE	GLZLM_LZHGE	GLCM_Homogeneity	stdValue
80	minValue	GLZLM_LZE	GLRLM_HGRE	SHAPE_Volume (mL	GLZLM_SZE	GLZLM_LZLGE
81	minValue	NGLDM_Busyness	GLZLM_SZE	GLCM_Homogeneity	GLZLM_LZE	NGLDM_Coarseness
82	GLCM_Homogeneity	GLRLM_HGRE	NGLDM_Busyness	GLZLM_SZE	minValue	GLRLM_LRHGE
83	minValue	NGLDM_Busyness	GLZLM_SZE	GLZLM_LZE	GLZLM_LZHGE	stdValue
84	minValue	GLZLM_SZE	GLZLM_LZE	GLZLM_LZLGE	HISTO_Energy	GLRLM_HGRE
85	GLZLM_LZE	minValue	GLRLM_HGRE	GLZLM_LZHGE	NGLDM_Busyness	GLZLM_SZE
86	minValue	GLZLM_LZE	GLZLM_SZE	GLZLM_LZHGE	HISTO_Energy	GLZLM_LZLGE
87	minValue	GLZLM_SZE	NGLDM_Busyness	GLRLM_HGRE	GLZLM_LZE	GLZLM_LZLGE
88	minValue	GLZLM_LZE	GLZLM_SZE	NGLDM_Busyness	GLRLM_LRHGE	GLZLM_LZLGE
89	minValue	GLZLM_LZE	GLZLM_LZLGE	GLZLM_LZHGE	GLCM_Homogeneity	GLRLM_HGRE
90	minValue	GLZLM_SZE	SHAPE_Volume (mL	GLZLM_LZLGE	GLZLM_LZE	GLZLM_ZLNU
91	minValue	GLZLM_LZHGE	GLZLM_LZE	GLZLM_SZE	GLRLM_LRHGE	GLCM_Homogeneity
92	GLZLM_SZE	GLZLM_LZE	NGLDM_Busyness	minValue	HISTO_Energy	GLZLM_SZLGE
93	minValue	GLZLM_LZLGE	NGLDM_Busyness	GLZLM_SZE	GLZLM_LZE	NGLDM_Coarseness
94	GLZLM_SZE	minValue	GLCM_Homogeneity	GLRLM_HGRE	GLZLM_LZE	SHAPE_Volume (mL)
95	GLZLM_SZE	minValue	GLZLM_LZLGE	stdValue	HISTO_Energy	GLZLM_LZE
96	GLZLM_LZE	GLZLM_LZLGE	minValue	HISTO_Energy	GLZLM_SZE	SHAPE_Volume (mL)
97	minValue	GLCM_Homogeneity	GLZLM_LZLGE	GLZLM_SZE	GLZLM_LZE	stdValue
98	minValue	NGLDM_Busyness	GLZLM_LZLGE	GLZLM_SZE	GLZLM_LZE	GLCM_Homogeneity
99	minValue	GLZLM_LZE	GLZLM_SZE	GLZLM_LZLGE	NGLDM_Coarseness	GLZLM_ZLNU
100	GLZLM_SZE	HISTO_Energy	GLZLM_LZLGE	GLZLM_LZE	GLCM_Homogeneity	minValue

Contrast-enhanced T1WI. Feature selector: LASSO

Cicle	Feature							
1	minValue	HISTO_Entropy_log	HISTO_Energy	NGLDM_Coarseness	GLZLM_SZE	GLZLM_HGZE	GLZLM_ZLNU	
2	stdValue	HISTO_Entropy_log	HISTO_Energy	GLRLM_RP	NGLDM_Coarseness	GLZLM_SZE	GLZLM_ZLNU	
3	minValue	HISTO_Entropy_log	NGLDM_Coarseness	GLZLM_SZE	GLZLM_ZLNU			
4	minValue	HISTO_Entropy_log	HISTO_Energy	GLRLM_SRHGE	GLRLM_RP	GLZLM_SZE	GLZLM_ZLNU	
5	stdValue	HISTO_Entropy_log	HISTO_Energy	GLRLM_SRHGE	GLRLM_RP	NGLDM_Coarseness	GLZLM_SZE	GLZLM_ZLNU
6	stdValue	HISTO_Energy	GLRLM_SRHGE	NGLDM_Coarseness	GLZLM_SZE	GLZLM_ZLNU		
7	HISTO_Energy	SHAPE_Volume (ml	GLCM_Homogeneity	NGLDM_Coarseness	GLZLM_SZE	GLZLM_ZLNU		
8	HISTO_Entropy_log	GLCM_Homogeneity	NGLDM_Coarseness	GLZLM_SZE	GLZLM_ZLNU			
9	minValue	HISTO_Entropy_log	NGLDM_Coarseness	GLZLM_SZE	GLZLM_ZLNU			
10	minValue	HISTO_Entropy_log	GLRLM_SRHGE	NGLDM_Coarseness	GLZLM_SZE	GLZLM_ZLNU		
11	HISTO_Entropy_log	HISTO_Energy	GLCM_Homogeneity	GLRLM_SRHGE	GLZLM_SZE	GLZLM_HGZE	GLZLM_ZLNU	
12	HISTO_Energy	NGLDM_Coarseness	GLZLM_SZE	GLZLM_ZLNU				
13	HISTO_Entropy_log	GLCM_Homogeneity	NGLDM_Coarseness	GLZLM_SZE	GLZLM_ZLNU			

14	HISTO_Entropy_log	NGLDM_Coarsenes	GLZLM_SZE	GLZLM_ZLNU				
15	HISTO_Energy	GLRLM_SRHGE	GLRLM_RP	NGLDM_Coarsenes	GLZLM_SZE	GLZLM_HGZE	GLZLM_ZLNU	
16	minValue	HISTO_Entropy_log	GLRLM_SRHGE	GLZLM_SZE	GLZLM_HGZE	GLZLM_ZLNU		
17	HISTO_Entropy_log	HISTO_Energy	GLRLM_SRHGE	GLZLM_SZE	GLZLM_ZLNU			
18	stdValue	HISTO_Entropy_log	HISTO_Energy	GLRLM_SRHGE	GLZLM_SZE	GLZLM_HGZE	GLZLM_ZLNU	
19	minValue	HISTO_Entropy_log	HISTO_Energy	GLCM_Homogeneity	NGLDM_Coarsenes	GLZLM_SZE	GLZLM_ZLNU	
20	HISTO_Energy	GLCM_Homogeneity	GLRLM_SRHGE	GLRLM_RP	GLZLM_SZE	GLZLM_ZLNU		
21	HISTO_Energy	GLRLM_RP	NGLDM_Coarsenes	GLZLM_SZE	GLZLM_ZLNU			
22	HISTO_Entropy_log	GLRLM_RP	NGLDM_Coarsenes	GLZLM_ZLNU				
23	minValue	stdValue	HISTO_Entropy_log	GLRLM_SRHGE	GLRLM_RP	NGLDM_Coarseness	GLZLM_ZLNU	
24	HISTO_Entropy_log	NGLDM_Coarsenes	GLZLM_SZE	GLZLM_HGZE	GLZLM_ZLNU			
25	stdValue	HISTO_Entropy_log	HISTO_Energy	GLRLM_SRHGE	GLZLM_SZE	GLZLM_HGZE	GLZLM_ZLNU	
26	minValue	HISTO_Energy	GLZLM_SZE	GLZLM_ZLNU				
27	HISTO_Entropy_log	GLZLM_SZE	GLZLM_ZLNU					
28	HISTO_Entropy_log	GLCM_Homogeneity	GLZLM_SZE	GLZLM_ZLNU				
29	minValue	stdValue	HISTO_Entropy_log	GLZLM_SZE	GLZLM_ZLNU			
30	stdValue	HISTO_Energy	GLRLM_RP	GLZLM_SZE	GLZLM_ZLNU			
31	minValue	HISTO_Entropy_log	HISTO_Energy	GLRLM_SRHGE	NGLDM_Coarsenes	GLZLM_SZE	GLZLM_ZLNU	
32	minValue	HISTO_Entropy_log	HISTO_Energy	NGLDM_Coarsenes	GLZLM_ZLNU			
33	stdValue	HISTO_Entropy_log	HISTO_Energy	GLCM_Homogeneity	GLRLM_RP	NGLDM_Coarseness	GLZLM_ZLNU	
34	HISTO_Energy	SHAPE_Volume (ml	GLRLM_SRHGE	GLRLM_RP	NGLDM_Coarsenes	GLZLM_SZE	GLZLM_ZLNU	
35	HISTO_Entropy_log	GLCM_Homogeneity	GLZLM_SZE	GLZLM_HGZE	GLZLM_ZLNU			
36	minValue	HISTO_Energy	GLRLM_SRHGE	GLRLM_RP	NGLDM_Busyness	GLZLM_SZE	GLZLM_ZLNU	
37	minValue	HISTO_Energy	GLCM_Homogeneity	NGLDM_Coarsenes	GLZLM_SZE	GLZLM_ZLNU		
38	HISTO_Entropy_log	HISTO_Energy	GLZLM_SZE	GLZLM_ZLNU				
39	minValue	HISTO_Energy	GLRLM_RP	NGLDM_Coarsenes	GLZLM_SZE	GLZLM_ZLNU		
40	minValue	HISTO_Entropy_log	HISTO_Energy	NGLDM_Coarsenes	GLZLM_SZE	GLZLM_ZLNU		
41	HISTO_Energy	GLCM_Homogeneity	GLRLM_SRHGE	GLRLM_RP	NGLDM_Coarsenes	GLZLM_SZE	GLZLM_ZLNU	
42	minValue	stdValue	HISTO_Entropy_log	HISTO_Energy	GLRLM_RP	GLZLM_ZLNU		
43	HISTO_Entropy_log	GLCM_Homogeneity	GLZLM_SZE	GLZLM_ZLNU				
44	minValue	HISTO_Energy	GLCM_Homogeneity	GLRLM_RP	NGLDM_Coarsenes	GLZLM_SZE	GLZLM_ZLNU	
45	minValue	stdValue	HISTO_Entropy_log	NGLDM_Coarsenes	GLZLM_SZE	GLZLM_HGZE	GLZLM_ZLNU	
46	minValue	HISTO_Entropy_log	HISTO_Energy	GLRLM_SRHGE	GLZLM_ZLNU			
47	stdValue	HISTO_Entropy_log	HISTO_Energy	GLRLM_SRHGE	NGLDM_Coarsenes	GLZLM_SZE	GLZLM_ZLNU	
48	minValue	HISTO_Energy	GLZLM_SZE	GLZLM_HGZE	GLZLM_ZLNU			
49	minValue	HISTO_Entropy_log	HISTO_Energy	GLRLM_SRHGE	NGLDM_Coarsenes	GLZLM_SZE	GLZLM_HGZE	GLZLM_ZLNU
50	minValue	HISTO_Entropy_log	HISTO_Energy	GLCM_Homogeneity	NGLDM_Coarsenes	GLZLM_SZE	GLZLM_ZLNU	
51	stdValue	HISTO_Energy	GLRLM_RP	GLZLM_SZE	GLZLM_ZLNU			
52	stdValue	HISTO_Entropy_log	HISTO_Energy	GLCM_Homogeneity	NGLDM_Coarsenes	GLZLM_SZE	GLZLM_ZLNU	
53	minValue	HISTO_Entropy_log	HISTO_Energy	GLRLM_SRHGE	GLRLM_RP	GLZLM_SZE	GLZLM_ZLNU	
54	minValue	stdValue	HISTO_Entropy_log	HISTO_Energy	GLRLM_RP	NGLDM_Coarseness	GLZLM_SZE	GLZLM_ZLNU
55	stdValue	HISTO_Entropy_log	GLRLM_RP	NGLDM_Coarsenes	GLZLM_ZLNU			
56	minValue	HISTO_Entropy_log	NGLDM_Coarsenes	GLZLM_SZE	GLZLM_ZLNU			
57	minValue	HISTO_Entropy_log	NGLDM_Coarsenes	GLZLM_SZE	GLZLM_ZLNU			
58	minValue	HISTO_Entropy_log	NGLDM_Coarsenes	GLZLM_SZE	GLZLM_ZLNU			
59	minValue	HISTO_Entropy_log	GLRLM_SRHGE	NGLDM_Coarsenes	GLZLM_SZE	GLZLM_ZLNU		
60	stdValue	HISTO_Energy	GLCM_Homogeneity	NGLDM_Coarsenes	GLZLM_SZE	GLZLM_ZLNU		
61	minValue	HISTO_Entropy_log	HISTO_Energy	GLZLM_SZE	GLZLM_ZLNU			
62	minValue	HISTO_Energy	NGLDM_Coarsenes	GLZLM_SZE	GLZLM_ZLNU			
63	minValue	HISTO_Entropy_log	HISTO_Energy	GLRLM_SRHGE	NGLDM_Coarsenes	GLZLM_SZE	GLZLM_ZLNU	
64	HISTO_Entropy_log	HISTO_Energy	GLRLM_RP	NGLDM_Coarsenes	GLZLM_SZE	GLZLM_ZLNU		
65	minValue	HISTO_Entropy_log	HISTO_Energy	GLCM_Homogeneity	NGLDM_Coarsenes	GLZLM_SZE	GLZLM_ZLNU	
66	minValue	HISTO_Entropy_log	HISTO_Energy	GLZLM_SZE	GLZLM_ZLNU			
67	HISTO_Energy	GLRLM_SRHGE	GLZLM_SZE	GLZLM_HGZE	GLZLM_ZLNU			
68	HISTO_Entropy_log	GLZLM_SZE	GLZLM_ZLNU					
69	HISTO_Energy	GLRLM_RP	GLZLM_SZE	GLZLM_ZLNU				

70	stdValue	HISTO_Entropy_log	HISTO_Energy	GLRLM_SRHGE	GLRLM_RP	NGLDM_Coarseness	GLZLM_ZLNU	
71	minValue	stdValue	HISTO_Entropy_log	HISTO_Energy	GLRLM_SRHGE	GLZLM_SZE	GLZLM_ZLNU	
72	stdValue	HISTO_Energy	NGLDM_Coarsenes	GLZLM_SZE	GLZLM_ZLNU			
73	minValue	HISTO_Entropy_log	HISTO_Energy	GLZLM_SZE	GLZLM_ZLNU			
74	minValue	HISTO_Entropy_log	HISTO_Energy	GLZLM_SZE	GLZLM_ZLNU			
75	minValue	HISTO_Entropy_log	HISTO_Energy	GLCM_Homogeneity	NGLDM_Coarsenes	GLZLM_SZE	GLZLM_ZLNU	
76	minValue	HISTO_Entropy_log	HISTO_Energy	GLRLM_SRHGE	NGLDM_Coarsenes	GLZLM_SZE	GLZLM_ZLNU	
77	stdValue	HISTO_Energy	GLRLM_SRHGE	GLZLM_SZE	GLZLM_ZLNU			
78	stdValue	HISTO_Energy	NGLDM_Coarsenes	GLZLM_SZE	GLZLM_ZLNU			
79	HISTO_Entropy_log	HISTO_Energy	GLRLM_SRHGE	NGLDM_Coarsenes	GLZLM_ZLNU			
80	HISTO_Energy	GLRLM_SRHGE	GLRLM_RP	GLZLM_SZE	GLZLM_ZLNU			
81	minValue	HISTO_Entropy_log	GLZLM_ZLNU					
82	minValue	HISTO_Entropy_log	HISTO_Energy	GLRLM_SRHGE	NGLDM_Coarsenes	GLZLM_SZE	GLZLM_ZLNU	
83	stdValue	SHAPE_Volume (ml	GLCM_Homogeneity	GLRLM_SRHGE	NGLDM_Coarsenes	GLZLM_SZE	GLZLM_HGZE	GLZLM_ZLNU
84	minValue	HISTO_Entropy_log	GLRLM_SRHGE	GLRLM_RP	NGLDM_Coarsenes	GLZLM_SZE	GLZLM_ZLNU	
85	minValue	stdValue	HISTO_Entropy_log	GLRLM_SRHGE	GLZLM_SZE	GLZLM_ZLNU		
86	stdValue	HISTO_Entropy_log	HISTO_Energy	GLZLM_SZE	GLZLM_ZLNU			
87	minValue	stdValue	HISTO_Energy	GLRLM_SRHGE	NGLDM_Coarsenes	GLZLM_SZE	GLZLM_ZLNU	
88	minValue	HISTO_Entropy_log	HISTO_Energy	GLCM_Homogeneity	NGLDM_Coarsenes	GLZLM_ZLNU		
89	minValue	HISTO_Entropy_log	HISTO_Energy	GLRLM_RP	NGLDM_Coarsenes	GLZLM_HGZE	GLZLM_ZLNU	
90	minValue	HISTO_Entropy_log	HISTO_Energy	GLRLM_SRHGE	GLZLM_SZE	GLZLM_HGZE	GLZLM_ZLNU	
91	HISTO_Entropy_log	HISTO_Energy	GLZLM_SZE	GLZLM_ZLNU				
92	minValue	stdValue	HISTO_Entropy_log	HISTO_Energy	NGLDM_Coarsenes	GLZLM_SZE	GLZLM_ZLNU	
93	minValue	HISTO_Entropy_log	GLRLM_SRHGE	NGLDM_Coarsenes	GLZLM_SZE	GLZLM_ZLNU		
94	HISTO_Entropy_log	HISTO_Energy	NGLDM_Coarsenes	GLZLM_SZE	GLZLM_ZLNU			
95	minValue	HISTO_Energy	GLZLM_SZE	GLZLM_ZLNU				
96	stdValue	HISTO_Entropy_log	HISTO_Energy	GLCM_Homogeneity	NGLDM_Coarsenes	GLZLM_SZE	GLZLM_ZLNU	
97	stdValue	HISTO_Energy	GLCM_Homogeneity	NGLDM_Coarsenes	GLZLM_SZE	GLZLM_ZLNU		
98	HISTO_Energy	GLCM_Homogeneity	GLRLM_RP	GLZLM_SZE	GLZLM_ZLNU			
99	minValue	HISTO_Energy	NGLDM_Coarsenes	GLZLM_SZE	GLZLM_ZLNU			
100	minValue	HISTO_Entropy_log	GLRLM_SRHGE	GLZLM_SZE	GLZLM_ZLNU			

T2WI. Feature selector: DC

Cicle	Feature					
1	GLCM_Homogeneity	GLRLM_SRE	GLRLM_RP	GLRLM_HGRE	GLRLM_LRHGE	GLRLM_SRLGE
2	GLRLM_SRLGE	GLRLM_LGRE	GLZLM_LGZE	GLCM_Homogeneity	GLRLM_LRLGE	GLRLM_SRE
3	NGLDM_Coarsenes	GLRLM_HGRE	GLRLM_LRHGE	GLRLM_RP	GLRLM_SRHGE	GLRLM_SRE
4	GLRLM_RP	GLRLM_SRE	GLCM_Homogeneity	NGLDM_Coarsenes	GLRLM_GLNU	GLRLM_SRLGE
5	GLCM_Homogeneity	GLRLM_RP	GLRLM_SRE	NGLDM_Coarsenes	GLRLM_SRLGE	GLRLM_LRHGE
6	GLRLM_RP	GLRLM_SRE	GLCM_Homogeneity	GLRLM_LRHGE	GLRLM_SRLGE	NGLDM_Coarseness
7	GLZLM_LGZE	GLRLM_HGRE	GLRLM_SRLGE	GLRLM_LGRE	GLRLM_SRHGE	GLZLM_SZHGE
8	GLCM_Homogeneity	GLRLM_RP	GLRLM_SRE	NGLDM_Coarsenes	GLCM_Dissimilarity	GLZLM_ZP
9	GLRLM_RP	GLRLM_SRE	GLCM_Homogeneity	NGLDM_Coarsenes	GLRLM_LRHGE	GLZLM_ZP
10	GLCM_Homogeneity	GLRLM_RP	GLRLM_SRE	GLRLM_SRLGE	GLRLM_LRHGE	GLZLM_LGZE
11	GLRLM_RP	GLRLM_SRE	GLCM_Homogeneity	GLRLM_LRHGE	GLRLM_HGRE	NGLDM_Coarseness
12	GLRLM_RP	GLRLM_SRE	GLCM_Homogeneity	GLRLM_LRHGE	GLRLM_HGRE	GLRLM_SRHGE
13	GLCM_Homogeneity	GLRLM_SRE	GLRLM_RP	GLRLM_SRLGE	GLRLM_LGRE	GLCM_Dissimilarity
14	GLRLM_RP	GLRLM_SRE	GLCM_Homogeneity	GLRLM_LRHGE	GLRLM_HGRE	GLRLM_SRHGE
15	GLRLM_SRE	GLRLM_RP	GLRLM_SRLGE	GLRLM_LGRE	GLCM_Homogeneity	GLRLM_LRHGE
16	GLRLM_HGRE	GLRLM_SRE	GLRLM_RP	GLCM_Homogeneity	GLRLM_LRHGE	GLRLM_SRHGE
17	GLZLM_LGZE	GLRLM_SRLGE	GLRLM_LGRE	GLZLM_SZLGE	GLRLM_HGRE	NGLDM_Coarseness
18	GLRLM_LRHGE	GLRLM_RP	NGLDM_Coarsenes	GLRLM_SRE	GLRLM_HGRE	GLRLM_SRLGE
19	NGLDM_Coarsenes	GLRLM_HGRE	GLRLM_SRLGE	GLZLM_LGZE	GLRLM_SRE	GLRLM_LRHGE
20	GLRLM_RP	GLRLM_SRE	NGLDM_Coarsenes	GLCM_Homogeneity	GLRLM_LRHGE	GLRLM_HGRE
21	GLCM_Homogeneity	GLRLM_RP	GLRLM_SRE	NGLDM_Coarsenes	GLRLM_LRHGE	GLRLM_SRLGE
22	GLRLM_HGRE	GLRLM_LRHGE	GLRLM_SRHGE	GLRLM_RP	GLRLM_SRE	GLRLM_SRLGE
23	GLRLM_RP	GLCM_Homogeneity	GLRLM_HGRE	GLRLM_SRE	GLRLM_LRHGE	GLRLM_SRHGE
24	GLRLM_RP	GLRLM_SRE	NGLDM_Coarsenes	GLCM_Homogeneity	GLZLM_ZP	GLRLM_SRLGE
25	GLCM_Homogeneity	GLRLM_SRE	GLRLM_RP	NGLDM_Coarsenes	GLRLM_SRLGE	GLZLM_LGZE
26	GLRLM_HGRE	GLRLM_LRHGE	GLRLM_SRHGE	GLRLM_RP	GLRLM_SRE	GLZLM_SZHGE
27	GLRLM_SRLGE	GLRLM_LGRE	GLZLM_LGZE	GLRLM_LRLGE	GLZLM_SZLGE	GLCM_Homogeneity
28	GLRLM_RP	GLRLM_SRE	GLCM_Homogeneity	GLRLM_SRLGE	GLZLM_LGZE	GLRLM_LGRE
29	GLRLM_HGRE	GLRLM_SRHGE	GLZLM_SZHGE	GLRLM_LRHGE	GLZLM_HGZE	HISTO_Skewness

30	GLRLM_SRLGE	GLRLM_LGRE	GLZLM_LGZE	GLRLM_HGRE	GLCM_Homogeneity	GLRLM_RP
31	GLRLM_RP	GLRLM_HGRE	GLRLM_SRE	NGLDM_Coarsenes	GLRLM_LRHGE	GLRLM_SRHGE
32	GLRLM_SRLGE	GLRLM_LGRE	GLRLM_HGRE	GLRLM_LRHGE	GLRLM_SRHGE	GLRLM_RP
33	GLRLM_SRLGE	GLRLM_RP	GLRLM_SRE	GLRLM_LGRE	GLRLM_HGRE	GLZLM_LGZE
34	GLRLM_RP	GLRLM_LRHGE	GLRLM_SRE	GLRLM_HGRE	GLCM_Homogeneity	GLRLM_SRHGE
35	GLRLM_RP	GLRLM_SRLGE	GLCM_Homogeneity	GLRLM_SRE	GLRLM_LRHGE	GLRLM_LGRE
36	GLZLM_LGZE	GLRLM_SRLGE	GLCM_Homogeneity	GLRLM_RP	GLRLM_LGRE	GLRLM_SRE
37	GLRLM_RP	GLRLM_SRE	NGLDM_Coarsenes	GLRLM_SRLGE	GLRLM_LRHGE	GLRLM_LGRE
38	GLRLM_SRE	NGLDM_Coarsenes	GLRLM_RP	GLRLM_SRLGE	GLRLM_LGRE	GLZLM_LGZE
39	GLRLM_RP	GLRLM_SRE	GLCM_Homogeneity	GLZLM_ZP	GLRLM_LRHGE	GLRLM_SRLGE
40	GLRLM_HGRE	GLRLM_SRHGE	GLRLM_LRHGE	GLZLM_HGZE	GLZLM_SZHGE	GLRLM_RP
41	GLRLM_SRLGE	GLRLM_LGRE	GLRLM_LRLGE	GLZLM_LGZE	GLRLM_HGRE	GLRLM_LRHGE
42	GLRLM_SRLGE	GLRLM_LGRE	GLCM_Homogeneity	GLRLM_SRE	GLRLM_RP	GLZLM_LGZE
43	GLRLM_HGRE	GLRLM_LRHGE	GLCM_Homogeneity	GLZLM_LGZE	GLRLM_SRLGE	GLRLM_SRHGE
44	GLRLM_HGRE	NGLDM_Coarsenes	GLRLM_RP	GLRLM_SRE	GLCM_Homogeneity	GLRLM_SRHGE
45	GLRLM_RP	GLCM_Homogeneity	GLRLM_SRLGE	GLRLM_SRE	GLRLM_LGRE	GLZLM_LGZE
46	GLRLM_SRLGE	GLZLM_LGZE	GLRLM_LGRE	GLCM_Homogeneity	GLZLM_SZHGE	GLRLM_HGRE
47	GLRLM_LRHGE	GLRLM_HGRE	GLRLM_SRHGE	GLRLM_RP	GLRLM_SRLGE	GLRLM_SRE
48	GLRLM_RP	GLRLM_SRE	GLCM_Homogeneity	GLRLM_LRHGE	GLZLM_ZP	GLRLM_LRE
49	GLRLM_SRLGE	GLRLM_LGRE	GLZLM_LGZE	GLRLM_LRLGE	GLZLM_SZLGE	GLRLM_HGRE
50	GLRLM_HGRE	GLRLM_LRHGE	GLRLM_RP	GLRLM_SRHGE	GLRLM_SRE	GLZLM_SZHGE
51	GLRLM_RP	GLRLM_SRE	NGLDM_Coarsenes	GLRLM_SRLGE	GLCM_Homogeneity	GLRLM_LRHGE
52	GLRLM_RP	GLRLM_SRE	GLCM_Homogeneity	NGLDM_Coarsenes	GLZLM_LGZE	GLRLM_SRLGE
53	GLRLM_RP	GLRLM_SRE	GLRLM_SRLGE	GLRLM_LGRE	NGLDM_Coarsenes	GLZLM_LGZE
54	GLZLM_LGZE	GLRLM_SRLGE	GLRLM_LGRE	GLRLM_LRHGE	NGLDM_Coarsenes	GLRLM_HGRE
55	GLRLM_LRHGE	GLRLM_HGRE	GLRLM_SRLGE	GLRLM_LGRE	GLRLM_RP	GLRLM_SRE
56	GLRLM_RP	GLRLM_SRLGE	GLRLM_LGRE	GLRLM_SRE	GLZLM_LGZE	GLRLM_LRHGE
57	GLRLM_RP	GLRLM_SRE	GLRLM_SRLGE	GLCM_Homogeneity	GLRLM_LGRE	NGLDM_Coarseness
58	GLRLM_SRLGE	GLRLM_LGRE	GLZLM_LGZE	GLRLM_RP	GLRLM_SRE	GLZLM_SZLGE
59	GLRLM_HGRE	GLRLM_LRHGE	GLCM_Homogeneity	GLRLM_SRHGE	GLRLM_RP	GLRLM_SRE
60	GLRLM_SRLGE	GLRLM_LGRE	GLZLM_LGZE	GLCM_Homogeneity	NGLDM_Coarsenes	GLRLM_SRE
61	NGLDM_Coarsenes	GLRLM_LRHGE	GLZLM_ZLNU	GLRLM_HGRE	GLZLM_LGZE	GLRLM_SRLGE
62	GLRLM_RP	GLRLM_SRE	NGLDM_Coarsenes	GLRLM_LRHGE	GLCM_Homogeneity	GLRLM_HGRE
63	GLRLM_RP	GLRLM_SRE	GLCM_Homogeneity	GLRLM_LRHGE	GLRLM_HGRE	GLRLM_SRLGE
64	GLRLM_SRE	GLCM_Homogeneity	GLRLM_RP	GLRLM_SRLGE	GLRLM_LGRE	GLZLM_LGZE
65	NGLDM_Coarsenes	GLRLM_RP	GLRLM_SRLGE	GLZLM_LGZE	GLRLM_SRE	GLRLM_LGRE
66	NGLDM_Coarsenes	GLRLM_RP	GLRLM_SRE	GLRLM_LRHGE	GLZLM_LGZE	GLCM_Homogeneity
67	GLRLM_RP	GLCM_Homogeneity	GLRLM_SRE	GLRLM_LRHGE	GLRLM_HGRE	NGLDM_Coarseness
68	GLRLM_HGRE	GLRLM_RP	GLRLM_SRE	GLRLM_LRHGE	GLRLM_SRHGE	GLCM_Homogeneity
69	GLRLM_RP	GLRLM_SRE	GLRLM_LRHGE	GLCM_Homogeneity	NGLDM_Coarsenes	GLRLM_HGRE
70	GLRLM_RP	GLRLM_SRE	GLCM_Homogeneity	NGLDM_Coarsenes	GLRLM_SRLGE	GLZLM_LGZE
71	GLCM_Homogeneity	GLRLM_SRE	GLRLM_RP	NGLDM_Coarsenes	GLRLM_SRLGE	GLRLM_LGRE
72	GLZLM_LGZE	GLRLM_SRLGE	GLRLM_HGRE	GLRLM_LGRE	GLRLM_SRHGE	GLZLM_HGZE
73	GLRLM_HGRE	GLRLM_LRHGE	GLZLM_LGZE	GLRLM_SRLGE	GLRLM_RP	GLZLM_SZHGE
74	GLRLM_HGRE	GLRLM_LRHGE	GLRLM_SRLGE	GLRLM_SRE	GLRLM_RP	GLCM_Homogeneity
75	GLCM_Homogeneity	GLRLM_RP	GLRLM_SRE	GLRLM_SRLGE	GLRLM_LGRE	GLZLM_ZP
76	GLRLM_HGRE	GLZLM_SZHGE	GLRLM_SRLGE	GLZLM_HGZE	GLRLM_LGRE	GLRLM_SRHGE
77	GLRLM_HGRE	GLRLM_LRHGE	GLRLM_SRLGE	GLRLM_LGRE	GLRLM_SRHGE	GLRLM_RP
78	GLRLM_RP	GLRLM_SRE	GLCM_Homogeneity	GLRLM_HGRE	GLRLM_LRHGE	NGLDM_Coarseness
79	GLRLM_HGRE	GLRLM_SRHGE	GLZLM_SZHGE	GLZLM_HGZE	GLRLM_LRHGE	GLRLM_SRLGE
80	GLRLM_SRLGE	GLRLM_LGRE	GLRLM_HGRE	GLZLM_LGZE	GLRLM_LRHGE	GLRLM_RP
81	GLRLM_SRLGE	GLRLM_LGRE	GLZLM_LGZE	GLRLM_LRLGE	GLZLM_SZLGE	NGLDM_Coarseness
82	GLCM_Homogeneity	GLRLM_RP	GLRLM_SRE	GLRLM_SRLGE	GLZLM_LGZE	GLRLM_LGRE
83	GLCM_Homogeneity	GLRLM_RP	GLRLM_SRLGE	GLRLM_SRE	GLRLM_LGRE	GLRLM_LRHGE
84	GLRLM_RP	GLRLM_SRE	NGLDM_Coarsenes	GLCM_Homogeneity	GLRLM_LRHGE	GLRLM_GLNU
85	GLRLM_RP	GLRLM_SRE	GLCM_Homogeneity	NGLDM_Coarsenes	GLRLM_SRLGE	GLZLM_LGZE
86	GLRLM_RP	GLRLM_SRE	GLCM_Homogeneity	GLRLM_LRHGE	NGLDM_Coarsenes	GLRLM_LRE
87	GLRLM_HGRE	GLRLM_LRHGE	GLRLM_SRHGE	GLRLM_SRE	GLRLM_RP	NGLDM_Coarseness
88	GLRLM_HGRE	GLRLM_LRHGE	GLRLM_SRHGE	GLRLM_RP	GLRLM_SRE	GLCM_Homogeneity
89	GLRLM_RP	GLRLM_SRE	GLCM_Homogeneity	GLRLM_GLNU	GLZLM_ZP	GLRLM_LRHGE
90	GLRLM_RP	GLCM_Homogeneity	GLRLM_SRE	GLRLM_SRLGE	GLRLM_LGRE	GLZLM_LGZE
91	GLRLM_HGRE	GLRLM_LRHGE	GLRLM_SRHGE	GLZLM_HGZE	GLZLM_SZHGE	GLRLM_SRLGE
92	GLZLM_SZHGE	GLRLM_HGRE	GLRLM_SRLGE	GLZLM_HGZE	GLRLM_LGRE	GLZLM_LGZE
93	GLCM_Homogeneity	GLRLM_SRE	GLRLM_RP	NGLDM_Coarsenes	GLRLM_SRLGE	GLZLM_LGZE
94	GLRLM_HGRE	GLRLM_SRHGE	GLZLM_SZHGE	GLZLM_HGZE	GLRLM_LRHGE	GLRLM_RP
95	GLZLM_LGZE	GLRLM_SRLGE	GLZLM_SZLGE	GLRLM_LGRE	NGLDM_Coarsenes	GLCM_Homogeneity
96	GLRLM_HGRE	GLRLM_SRHGE	GLZLM_HGZE	GLZLM_SZHGE	GLRLM_LRHGE	HISTO_Skewness
97	GLRLM_LRHGE	GLRLM_HGRE	GLZLM_LGZE	GLRLM_SRLGE	GLRLM_RP	GLRLM_LGRE
98	GLRLM_RP	GLRLM_SRE	GLCM_Homogeneity	NGLDM_Coarsenes	GLZLM_ZP	GLRLM_GLNU
99	GLRLM_SRLGE	GLCM_Homogeneity	GLRLM_LRLGE	GLRLM_HGRE	GLRLM_LGRE	GLRLM_LRHGE
100	GLRLM_SRLGE	GLRLM_LGRE	GLRLM_HGRE	GLRLM_LRHGE	NGLDM_Coarsenes	GLZLM_LGZE

T2WI. Feature selector: RFs

Cicle	Feature					
1	minValue	GLZLM_LZE	GLZLM_LZHGE	GLRLM_GLNU	GLZLM_SZHGE	GLZLM_SZE

2	GLZLM_LZE	GLRLM_SRLGE	GLRLM_LRLGE	GLZLM_LZHGE	GLZLM_SZE	GLZLM_LGZE
3	GLRLM_HGRE	GLZLM_LZHGE	GLZLM_HGZE	GLZLM_SZHGE	GLZLM_LZE	minValue
4	GLZLM_LZHGE	GLRLM_SRLGE	GLZLM_GLNU	GLZLM_LZE	GLRLM_GLNU	GLRLM_RP
5	GLZLM_LZE	GLZLM_SZE	minValue	GLZLM_LZHGE	GLRLM_LGRE	GLZLM_ZP
6	GLZLM_LZE	GLZLM_LZHGE	NGLDM_Coarsenes	GLRLM_SRLGE	GLZLM_SZE	GLZLM_ZP
7	GLZLM_LZHGE	GLRLM_SRLGE	GLZLM_SZE	minValue	GLZLM_SZHGE	GLZLM_SZLGE
8	GLZLM_LZE	minValue	GLZLM_LZHGE	GLRLM_SRE	GLCM_Homogeneity	NGLDM_Coarseness
9	GLZLM_LZHGE	GLZLM_LZE	GLRLM_SRLGE	GLRLM_LRE	GLRLM_GLNU	GLZLM_LZLGE
10	GLZLM_LZE	GLCM_Homogeneity	GLZLM_LZHGE	GLZLM_SZE	GLZLM_SZHGE	GLZLM_HGZE
11	GLZLM_LZHGE	GLRLM_HGRE	GLCM_Homogeneity	GLRLM_GLNU	GLZLM_SZHGE	GLRLM_LRLGE
12	GLRLM_HGRE	minValue	GLZLM_LZHGE	GLZLM_HGZE	GLZLM_SZE	GLZLM_LZLGE
13	GLZLM_LZE	GLZLM_LZHGE	GLRLM_SRLGE	minValue	GLZLM_SZHGE	GLZLM_SZE
14	GLZLM_LZE	GLZLM_LZHGE	GLRLM_HGRE	GLZLM_HGZE	GLRLM_SRLGE	GLZLM_SZE
15	GLRLM_SRLGE	GLRLM_HGRE	GLZLM_LZHGE	GLZLM_SZE	GLZLM_LZE	GLZLM_SZHGE
16	GLRLM_HGRE	GLZLM_LZHGE	GLZLM_SZE	GLZLM_HGZE	GLZLM_SZHGE	GLRLM_GLNU
17	GLZLM_SZE	GLRLM_LRLGE	GLZLM_LZHGE	minValue	GLRLM_HGRE	GLRLM_SRLGE
18	GLZLM_LZHGE	GLRLM_SRLGE	GLRLM_HGRE	NGLDM_Coarsenes	GLZLM_SZHGE	GLZLM_SZE
19	GLRLM_SRLGE	GLRLM_HGRE	GLZLM_ZLNU	GLZLM_LZE	GLZLM_HGZE	GLRLM_LRLGE
20	GLZLM_LZE	GLZLM_LZHGE	GLRLM_HGRE	minValue	GLZLM_SZE	GLRLM_RP
21	GLZLM_LZE	minValue	GLZLM_HGZE	GLCM_Homogeneity	GLZLM_SZHGE	GLZLM_LZHGE
22	GLRLM_HGRE	GLZLM_LZHGE	GLZLM_LZE	GLZLM_SZE	GLRLM_SRHGE	GLRLM_SRE
23	GLRLM_HGRE	GLZLM_LZHGE	GLZLM_LZE	GLRLM_SRLGE	GLZLM_HGZE	GLZLM_SZHGE
24	GLZLM_LZHGE	NGLDM_Coarsenes	GLZLM_LZE	GLZLM_GLNU	minValue	GLRLM_LRE
25	minValue	GLZLM_SZE	GLZLM_LZE	GLCM_Homogeneity	GLRLM_GLNU	GLZLM_LZHGE
26	GLRLM_HGRE	GLZLM_SZE	GLZLM_LZHGE	GLZLM_HGZE	minValue	GLRLM_GLNU
27	GLRLM_SRLGE	GLZLM_SZE	GLZLM_LZHGE	minValue	GLZLM_SZLGE	GLZLM_HGZE
28	GLZLM_LZE	GLZLM_SZE	GLZLM_LGZE	minValue	GLZLM_SZHGE	GLRLM_LRLGE
29	GLRLM_HGRE	GLZLM_SZE	GLZLM_HGZE	GLZLM_LZHGE	HISTO_Skewness	GLZLM_LZE
30	GLRLM_SRLGE	GLZLM_LZHGE	GLRLM_LRLGE	GLRLM_LGRE	GLZLM_SZHGE	GLZLM_LGZE
31	GLZLM_LZHGE	GLRLM_HGRE	NGLDM_Coarsenes	GLRLM_LRLGE	GLRLM_RP	minValue
32	GLRLM_SRLGE	GLZLM_LZHGE	minValue	GLRLM_LRHGE	GLZLM_SZHGE	GLRLM_HGRE
33	GLRLM_SRLGE	GLZLM_LZHGE	minValue	GLZLM_LGZE	GLZLM_HGZE	GLZLM_SZE
34	GLZLM_LZHGE	GLZLM_LZE	GLRLM_HGRE	GLRLM_LGRE	minValue	GLRLM_SRE
35	GLZLM_LZHGE	GLZLM_LZE	GLZLM_HGZE	minValue	GLCM_Homogeneity	NGLDM_Coarseness
36	GLRLM_SRLGE	HISTO_Energy	GLRLM_RP	GLRLM_GLNU	GLZLM_LGZE	GLZLM_SZE
37	GLZLM_LZHGE	GLRLM_SRLGE	GLRLM_HGRE	GLZLM_LZE	minValue	GLZLM_ZP
38	GLRLM_SRLGE	minValue	GLRLM_LRLGE	GLZLM_LZE	GLRLM_HGRE	NGLDM_Coarseness
39	GLZLM_LZHGE	GLZLM_LZE	GLRLM_SRLGE	GLRLM_LRE	minValue	GLZLM_ZP
40	GLRLM_HGRE	GLZLM_LZHGE	GLZLM_SZHGE	GLZLM_LZE	GLRLM_RP	GLZLM_HGZE
41	GLZLM_LZHGE	GLRLM_HGRE	GLRLM_LGRE	GLZLM_SZE	GLRLM_SRLGE	minValue
42	GLZLM_LZHGE	GLZLM_HGZE	GLRLM_HGRE	GLRLM_LRE	GLCM_Homogeneity	GLRLM_SRLGE
43	GLRLM_SRLGE	GLZLM_LZE	GLRLM_LRLGE	GLZLM_LZHGE	GLRLM_HGRE	GLZLM_GLNU
44	GLZLM_LZHGE	GLRLM_LRE	GLZLM_HGZE	GLRLM_RLNU	GLZLM_SZHGE	NGLDM_Coarseness
45	GLZLM_LZE	GLZLM_HGZE	GLZLM_LZHGE	GLRLM_LRLGE	GLRLM_SRLGE	GLZLM_SZHGE
46	GLZLM_SZHGE	GLZLM_SZE	GLZLM_LGZE	GLRLM_SRLGE	GLRLM_LRLGE	minValue
47	GLRLM_HGRE	GLZLM_LZHGE	NGLDM_Coarsenes	GLZLM_SZE	GLZLM_HGZE	NGLDM_Busyness
48	GLZLM_LZE	minValue	GLRLM_SRLGE	GLZLM_LZHGE	GLCM_Homogeneity	GLRLM_GLNU
49	GLRLM_SRLGE	HISTO_Skewness	GLZLM_HGZE	GLRLM_LRLGE	minValue	GLZLM_LZHGE
50	GLZLM_HGZE	GLZLM_LZHGE	GLRLM_HGRE	GLCM_Correlation	GLRLM_GLNU	minValue
51	GLRLM_GLNU	GLRLM_RLNU	GLZLM_GLNU	GLRLM_HGRE	GLZLM_HGZE	GLRLM_SRLGE
52	GLRLM_GLNU	NGLDM_Coarsenes	GLRLM_SRLGE	GLZLM_HGZE	GLZLM_ZLNU	GLZLM_LZE
53	GLZLM_LZE	GLZLM_LZHGE	GLZLM_ZP	GLZLM_SZE	GLRLM_LRE	GLRLM_RP
54	GLRLM_HGRE	GLRLM_SRLGE	GLRLM_LRLGE	GLZLM_HGZE	GLRLM_RLNU	GLZLM_LZE
55	GLZLM_LZHGE	GLRLM_HGRE	GLZLM_HGZE	GLZLM_SZE	HISTO_Skewness	GLRLM_LRLGE
56	GLZLM_LZHGE	GLRLM_HGRE	GLZLM_LZE	GLZLM_LGZE	GLRLM_GLNU	GLRLM_SRLGE
57	GLRLM_GLNU	minValue	GLZLM_SZHGE	GLZLM_ZP	GLZLM_LZHGE	GLZLM_LZE
58	GLZLM_LZHGE	GLZLM_LZE	GLRLM_SRLGE	minValue	GLZLM_HGZE	GLZLM_SZHGE
59	GLZLM_HGZE	GLRLM_GLNU	GLRLM_SRLGE	GLRLM_HGRE	GLZLM_SZHGE	GLZLM_GLNU
60	GLRLM_SRLGE	GLZLM_LZHGE	GLZLM_LZE	GLZLM_HGZE	GLRLM_LRLGE	GLRLM_GLNU
61	GLRLM_GLNU	GLRLM_HGRE	GLZLM_LZE	GLZLM_ZLNU	GLRLM_LRHGE	GLZLM_GLNU
62	GLZLM_LZHGE	GLZLM_SZE	GLZLM_SZHGE	GLZLM_LZE	NGLDM_Coarsenes	GLRLM_HGRE
63	GLZLM_LZE	GLZLM_LZHGE	GLZLM_LZLGE	GLRLM_HGRE	minValue	GLZLM_SZHGE
64	GLZLM_LZHGE	GLZLM_LZE	GLRLM_SRLGE	GLRLM_LRHGE	GLCM_Homogeneity	GLRLM_HGRE
65	GLZLM_LZHGE	GLRLM_GLNU	GLZLM_LZE	GLRLM_SRLGE	minValue	GLRLM_LRLGE
66	GLZLM_LZE	GLRLM_GLNU	GLZLM_LZHGE	GLRLM_RLNU	GLZLM_HGZE	GLZLM_LGZE
67	GLZLM_LZHGE	GLRLM_HGRE	GLCM_Homogeneity	minValue	GLRLM_LRE	GLZLM_LZE
68	GLZLM_LZHGE	GLRLM_HGRE	GLZLM_HGZE	GLZLM_SZHGE	GLZLM_ZLNU	GLZLM_SZE
69	GLZLM_LZHGE	minValue	GLRLM_HGRE	GLZLM_LZE	GLZLM_LGZE	GLRLM_SRE
70	GLCM_Homogeneity	minValue	GLZLM_LZE	GLZLM_SZE	GLRLM_SRLGE	GLZLM_GLNU
71	GLZLM_LZHGE	GLZLM_ZLNU	GLZLM_LZE	GLRLM_LRLGE	GLZLM_SZE	GLZLM_LGZE
72	GLRLM_SRLGE	GLZLM_SZHGE	GLRLM_GLNU	GLRLM_LRLGE	GLZLM_LZHGE	GLZLM_HGZE
73	GLRLM_HGRE	GLZLM_SZE	GLZLM_LZHGE	minValue	GLRLM_LRLGE	GLZLM_LZE
74	GLZLM_LZHGE	GLRLM_HGRE	GLZLM_HGZE	GLZLM_SZE	GLRLM_LGRE	GLRLM_SRLGE
75	GLZLM_LZE	GLZLM_LZHGE	GLCM_Homogeneity	GLZLM_SZHGE	GLRLM_GLNU	GLZLM_ZP
76	GLRLM_HGRE	GLRLM_SRLGE	GLZLM_SZHGE	GLZLM_HGZE	GLZLM_LZHGE	minValue
77	GLRLM_LRLGE	GLRLM_SRLGE	GLZLM_LZHGE	GLZLM_SZE	GLRLM_HGRE	GLZLM_ZLNU

78	GLZLM_LZHGE	GLRLM_SRLGE	GLRLM_GLNU	GLRLM_HGRE	minValue	GLZLM_HGZE
79	GLRLM_HGRE	GLZLM_SZHGE	GLZLM_HGZE	GLZLM_LZHGE	GLZLM_SZE	GLRLM_LRHGE
80	GLZLM_SZHGE	GLRLM_SRLGE	GLRLM_HGRE	GLZLM_LZHGE	GLZLM_LZE	GLZLM_SZE
81	GLZLM_GLNU	GLZLM_LZE	GLZLM_LZHGE	GLZLM_SZE	GLCM_Homogeneit	GLRLM_RLNU
82	minValue	GLZLM_LZHGE	GLZLM_SZHGE	GLZLM_LZE	GLRLM_SRLGE	GLZLM_HGZE
83	GLZLM_LZHGE	GLRLM_SRLGE	GLRLM_LRLGE	GLZLM_LZE	GLCM_Homogeneit	GLRLM_LGRE
84	GLZLM_LZHGE	GLZLM_LZE	GLZLM_GLNU	GLRLM_LRLGE	GLRLM_SRLGE	HISTO_Energy
85	GLZLM_LZE	GLRLM_SRLGE	GLZLM_ZLNU	NGLDM_Coarsenes	GLZLM_SZE	GLCM_Homogeneity
86	GLRLM_RLNU	GLZLM_LZHGE	minValue	GLRLM_SRE	GLRLM_GLNU	GLRLM_HGRE
87	GLZLM_LZHGE	GLRLM_HGRE	GLZLM_HGZE	HISTO_Skewness	GLRLM_GLNU	GLZLM_SZE
88	GLZLM_LZHGE	GLZLM_HGZE	GLRLM_HGRE	GLZLM_SZE	GLRLM_GLNU	HISTO_Skewness
89	GLZLM_LZE	GLZLM_LZHGE	HISTO_Energy	GLRLM_SRE	GLZLM_ZP	GLCM_Homogeneity
90	GLRLM_LRLGE	GLZLM_SZE	GLZLM_LZHGE	GLRLM_SRE	GLZLM_LZE	NGLDM_Coarsenes
91	GLRLM_HGRE	GLZLM_HGZE	GLRLM_LRLGE	GLZLM_LZHGE	GLRLM_LRE	minValue
92	GLZLM_SZHGE	GLRLM_SRLGE	GLZLM_LZHGE	GLCM_Homogeneit	GLZLM_SZE	GLRLM_LGRE
93	GLRLM_SRLGE	GLZLM_SZE	GLZLM_LZE	NGLDM_Coarsenes	GLZLM_LZHGE	GLZLM_HGZE
94	GLZLM_SZHGE	GLRLM_GLNU	GLZLM_LZE	GLZLM_LZHGE	GLRLM_LRLGE	GLZLM_SZE
95	GLRLM_SRLGE	minValue	GLZLM_ZP	GLZLM_LZHGE	GLZLM_SZLGE	GLCM_Homogeneity
96	GLRLM_HGRE	HISTO_Skewness	GLZLM_LZHGE	GLZLM_SZHGE	GLZLM_LZE	GLCM_Homogeneity
97	GLZLM_LZE	GLZLM_LZHGE	GLRLM_HGRE	GLZLM_ZLNU	GLCM_Correlation	GLRLM_LGRE
98	GLZLM_LZHGE	GLZLM_LZE	GLZLM_ZLNU	GLRLM_SRLGE	GLCM_Homogeneit	GLZLM_ZP
99	GLRLM_LRLGE	minValue	GLRLM_SRLGE	GLCM_Homogeneit	HISTO_Skewness	GLRLM_LGRE
100	GLRLM_SRLGE	GLZLM_LZHGE	GLRLM_HGRE	GLRLM_LRLGE	GLZLM_SZHGE	GLRLM_SRHGE

T2WI. Feature selector: LASSO

Cicle	Feature					
1	GLCM_Homogeneit	GLCM_Correlation	GLRLM_HGRE	GLRLM_SRLGE	GLZLM_SZHGE	GLZLM_ZLNU
2	GLCM_Homogeneit	GLRLM_SRLGE	NGLDM_Coarsenes	GLZLM_LGZE	GLZLM_SZHGE	GLZLM_ZLNU
3	GLCM_Correlation	GLRLM_HGRE	GLRLM_RP	GLZLM_SZHGE	GLZLM_ZLNU	
4	GLCM_Homogeneit	GLRLM_HGRE	GLRLM_SRLGE	NGLDM_Coarsenes	GLZLM_SZLGE	GLZLM_ZLNU
5	GLCM_Homogeneit	GLCM_Correlation	GLRLM_HGRE	GLRLM_SRLGE	NGLDM_Coarseness	
6	GLCM_Homogeneit	GLCM_Correlation	GLRLM_HGRE	GLRLM_SRLGE	GLRLM_RP	NGLDM_Coarsenes
	GLZLM_SZLGE	GLZLM_ZLNU				
7	GLCM_Homogeneit	GLCM_Correlation	GLRLM_RP	NGLDM_Coarsenes	GLZLM_LGZE	GLZLM_SZHGE
	GLZLM_ZLNU					
8	GLCM_Homogeneit	GLCM_Correlation	GLRLM_HGRE	GLRLM_SRLGE	NGLDM_Coarsenes	GLZLM_ZLNU
9	GLCM_Homogeneit	GLCM_Correlation	GLRLM_SRE	GLRLM_HGRE	GLRLM_RP	NGLDM_Coarsenes
	GLZLM_SZLGE	GLZLM_ZLNU				
10	GLCM_Homogeneit	GLRLM_SRLGE	GLZLM_SZHGE	GLZLM_ZLNU		
11	GLCM_Homogeneit	GLRLM_RP	NGLDM_Coarsenes	GLZLM_SZLGE	GLZLM_SZHGE	GLZLM_ZLNU
12	GLCM_Homogeneit	GLCM_Correlation	GLRLM_HGRE	GLRLM_RP		
13	GLCM_Homogeneit	GLCM_Correlation	GLRLM_SRLGE	GLZLM_SZLGE	GLZLM_SZHGE	GLZLM_ZLNU
14	GLCM_Homogeneit	GLRLM_HGRE	GLRLM_SRLGE	GLRLM_RP	GLZLM_SZHGE	GLZLM_ZLNU
15	GLCM_Homogeneit	GLRLM_HGRE	GLRLM_SRLGE	GLRLM_RP	NGLDM_Coarsenes	GLZLM_SZHGE
16	GLCM_Homogeneit	GLCM_Correlation	GLRLM_HGRE	GLRLM_RP	GLZLM_SZHGE	
17	GLCM_Homogeneit	GLCM_Correlation	GLRLM_SRLGE	GLZLM_SZLGE	GLZLM_SZHGE	GLZLM_ZLNU
18	GLRLM_HGRE	GLRLM_SRLGE	GLRLM_RP	GLZLM_SZHGE	GLZLM_ZLNU	
19	GLCM_Homogeneit	GLRLM_SRE	GLRLM_HGRE	NGLDM_Coarsenes	GLZLM_SZLGE	GLZLM_SZHGE
	GLZLM_ZLNU					
20	GLCM_Homogeneit	GLCM_Correlation	GLRLM_HGRE	GLRLM_SRLGE	GLRLM_RP	NGLDM_Coarsenes
21	GLCM_Homogeneit	GLRLM_RP	NGLDM_Coarsenes	GLZLM_SZHGE	GLZLM_ZLNU	
22	GLRLM_SRE	GLRLM_HGRE	GLRLM_SRLGE	GLRLM_RP	NGLDM_Coarsenes	GLZLM_ZLNU
23	GLCM_Homogeneit	GLCM_Correlation	GLRLM_HGRE	NGLDM_Coarsenes	GLZLM_ZLNU	
24	GLCM_Homogeneit	GLCM_Correlation	GLRLM_HGRE	GLRLM_SRLGE	GLRLM_RP	NGLDM_Coarsenes
	GLZLM_SZHGE	GLZLM_ZLNU				
25	GLCM_Homogeneit	NGLDM_Coarsenes	GLZLM_LGZE	GLZLM_SZHGE	GLZLM_ZLNU	
26	GLCM_Correlation	GLRLM_SRE	GLRLM_HGRE	GLRLM_RP	GLZLM_ZLNU	
27	GLCM_Homogeneit	GLCM_Correlation	GLRLM_HGRE	GLRLM_SRLGE	GLZLM_ZLNU	
28	GLRLM_RP	GLZLM_LGZE	GLZLM_SZHGE	GLZLM_ZLNU		
29	GLCM_Homogeneit	GLRLM_SRE	GLZLM_SZE	GLZLM_SZHGE	GLZLM_ZLNU	
30	GLCM_Homogeneit	GLCM_Correlation	GLRLM_SRLGE	NGLDM_Coarsenes	GLZLM_SZLGE	GLZLM_SZHGE
	GLZLM_ZLNU					
31	GLCM_Homogeneit	GLRLM_SRE	GLRLM_HGRE	NGLDM_Coarsenes	GLZLM_SZHGE	GLZLM_ZLNU
32	GLRLM_HGRE	GLRLM_RP	GLZLM_LGZE	GLZLM_SZLGE		
33	GLCM_Homogeneit	GLCM_Dissimilarity	GLRLM_HGRE	NGLDM_Coarsenes	GLZLM_ZLNU	
34	GLCM_Homogeneit	GLRLM_SRE	GLRLM_HGRE	GLRLM_SRLGE	GLRLM_RP	NGLDM_Coarsenes
35	GLCM_Homogeneit	GLCM_Correlation	GLRLM_HGRE	GLRLM_SRLGE	GLZLM_SZLGE	
36	GLCM_Homogeneit	GLZLM_LGZE	GLZLM_SZLGE	GLZLM_SZHGE	GLZLM_ZLNU	
37	GLRLM_HGRE	GLRLM_SRLGE	GLRLM_RP	NGLDM_Coarsenes	GLZLM_ZLNU	
38	GLCM_Homogeneit	GLRLM_SRE	GLRLM_SRLGE	NGLDM_Coarsenes	GLZLM_SZHGE	GLZLM_ZLNU
39	GLCM_Homogeneit	GLCM_Correlation	GLRLM_HGRE	GLRLM_SRLGE	GLRLM_RP	
40	GLCM_Homogeneit	GLCM_Correlation	GLRLM_HGRE	NGLDM_Coarsenes	GLZLM_SZHGE	
41	GLCM_Homogeneit	GLCM_Correlation	GLRLM_HGRE	GLRLM_SRLGE	GLZLM_ZLNU	
42	minValue	GLCM_Homogeneit	GLCM_Correlation	GLRLM_HGRE	GLRLM_RP	GLZLM_LGZE
	GLZLM_SZHGE					

43	GLCM_Homogeneity	GLCM_Dissimilarity	GLRLM_HGRE	NGLDM_Coarseness	GLZLM_SZLGE	GLZLM_ZLNU
44	GLCM_Homogeneity	GLCM_Correlation	GLRLM_HGRE	GLRLM_RP	NGLDM_Coarseness	GLZLM_SZHGE
	GLZLM_ZLNU					
45	GLCM_Homogeneity	GLCM_Correlation	GLRLM_SRLGE	NGLDM_Coarseness	GLZLM_SZLGE	GLZLM_SZHGE
46	GLCM_Homogeneity	GLCM_Correlation	NGLDM_Coarseness	GLZLM_SZE	GLZLM_LGZE	GLZLM_SZHGE
47	GLRLM_HGRE	GLRLM_SRLGE	GLRLM_RP	NGLDM_Coarseness	GLZLM_ZLNU	
48	GLCM_Homogeneity	GLCM_Correlation	GLRLM_HGRE	GLRLM_RP	NGLDM_Coarseness	GLZLM_LGZE
49	GLCM_Homogeneity	GLCM_Dissimilarity	GLRLM_HGRE	GLRLM_SRLGE	NGLDM_Coarseness	GLZLM_SZLGE
	GLZLM_SZHGE	GLZLM_ZLNU				
50	GLCM_Homogeneity	GLCM_Correlation	GLRLM_HGRE	GLRLM_RP	GLZLM_SZHGE	GLZLM_ZLNU
51	GLCM_Homogeneity	GLRLM_SRE	GLRLM_HGRE	GLRLM_SRLGE	NGLDM_Coarseness	GLZLM_SZLGE
	GLZLM_ZLNU					
52	GLCM_Homogeneity	NGLDM_Coarseness	GLZLM_SZLGE	GLZLM_SZHGE	GLZLM_ZLNU	
53	GLCM_Homogeneity	GLRLM_SRE	NGLDM_Coarseness	GLZLM_SZLGE	GLZLM_SZHGE	GLZLM_ZLNU
54	GLRLM_HGRE	GLRLM_RP	NGLDM_Coarseness	GLZLM_LGZE	GLZLM_SZHGE	GLZLM_ZLNU
55	GLRLM_HGRE	GLRLM_SRLGE	GLRLM_RP	NGLDM_Coarseness	GLZLM_LGZE	
56	GLRLM_HGRE	GLRLM_RP	GLZLM_SZLGE			
57	GLCM_Homogeneity	GLRLM_RP	GLZLM_SZLGE	GLZLM_SZHGE	GLZLM_ZLNU	
58	GLCM_Correlation	GLRLM_HGRE	GLRLM_SRLGE	GLRLM_RP	GLZLM_SZLGE	GLZLM_SZHGE
	GLZLM_ZLNU					
59	GLCM_Homogeneity	GLCM_Dissimilarity	GLRLM_HGRE	NGLDM_Coarseness	GLZLM_ZLNU	
60	GLCM_Homogeneity	GLCM_Correlation	GLRLM_HGRE	GLRLM_SRLGE	NGLDM_Coarseness	GLZLM_HGZE
	GLZLM_ZLNU					
61	minValue	GLCM_Homogeneity	GLCM_Correlation	GLRLM_HGRE	GLRLM_SRLGE	NGLDM_Coarseness
	GLZLM_ZLNU					
62	GLCM_Homogeneity	GLRLM_HGRE	GLRLM_SRLGE	GLRLM_RP	NGLDM_Coarseness	GLZLM_SZHGE
	GLZLM_ZLNU					
63	GLCM_Homogeneity	GLRLM_HGRE	GLRLM_SRLGE	GLRLM_RP	GLZLM_SZHGE	GLZLM_ZLNU
64	GLCM_Homogeneity	GLRLM_HGRE	GLRLM_SRLGE	NGLDM_Coarseness	GLZLM_ZLNU	
65	GLCM_Correlation	GLRLM_HGRE	GLRLM_RP	NGLDM_Coarseness	GLZLM_LGZE	GLZLM_ZLNU
66	GLCM_Homogeneity	GLRLM_RP	NGLDM_Coarseness	GLZLM_SZHGE	GLZLM_ZLNU	
67	GLCM_Homogeneity	GLCM_Correlation	GLRLM_HGRE	NGLDM_Coarseness	GLZLM_SZLGE	GLZLM_ZLNU
68	GLCM_Homogeneity	GLCM_Correlation	GLRLM_HGRE	GLRLM_RP	NGLDM_Coarseness	GLZLM_LGZE
	GLZLM_SZHGE					
69	GLCM_Homogeneity	GLRLM_HGRE	GLRLM_RP	NGLDM_Coarseness	GLZLM_SZE	GLZLM_ZLNU
70	GLCM_Homogeneity	GLCM_Correlation	GLRLM_HGRE	GLRLM_RP	NGLDM_Coarseness	GLZLM_ZLNU
71	GLCM_Homogeneity	GLRLM_SRLGE	NGLDM_Coarseness	GLZLM_SZHGE	GLZLM_ZLNU	
72	GLCM_Homogeneity	GLCM_Correlation	GLRLM_SRLGE	NGLDM_Coarseness	GLZLM_SZLGE	GLZLM_SZHGE
	GLZLM_ZLNU					
73	GLCM_Correlation	GLRLM_RP	GLZLM_SZHGE	GLZLM_ZLNU		
74	GLCM_Homogeneity	GLRLM_SRLGE	GLRLM_RP	NGLDM_Coarseness	GLZLM_SZHGE	GLZLM_ZLNU
75	GLCM_Homogeneity	GLCM_Correlation	GLRLM_HGRE	GLZLM_SZLGE	GLZLM_SZHGE	
76	GLCM_Homogeneity	GLCM_Correlation	GLRLM_HGRE	GLZLM_SZHGE	GLZLM_ZLNU	
77	GLCM_Homogeneity	GLRLM_HGRE	GLRLM_SRLGE	NGLDM_Coarseness	GLZLM_LGZE	GLZLM_SZHGE
78	GLCM_Homogeneity	GLCM_Correlation	GLRLM_HGRE	GLRLM_SRLGE	NGLDM_Coarseness	GLZLM_SZHGE
	GLZLM_ZLNU					
79	GLCM_Homogeneity	GLCM_Correlation	GLRLM_RP	NGLDM_Coarseness	GLZLM_SZHGE	GLZLM_ZLNU
80	GLCM_Homogeneity	GLRLM_RP	GLZLM_SZLGE	GLZLM_SZHGE		
81	GLCM_Homogeneity	GLRLM_HGRE	GLRLM_SRLGE	GLRLM_RP	NGLDM_Coarseness	GLZLM_SZLGE
	GLZLM_ZLNU					
82	GLCM_Homogeneity	GLCM_Correlation	GLRLM_SRLGE	GLZLM_SZHGE		
83	GLCM_Homogeneity	GLCM_Correlation	GLCM_Dissimilarity	GLRLM_HGRE	GLRLM_SRLGE	GLRLM_RP
	GLZLM_SZLGE	GLZLM_ZLNU				
84	GLCM_Homogeneity	GLRLM_HGRE	GLRLM_RP	NGLDM_Coarseness	GLZLM_SZLGE	GLZLM_SZHGE
	GLZLM_ZLNU					
85	GLCM_Homogeneity	GLRLM_RP	NGLDM_Coarseness	GLZLM_SZHGE	GLZLM_ZLNU	
86	GLCM_Homogeneity	GLRLM_HGRE	GLRLM_SRLGE	GLRLM_RP	GLZLM_ZLNU	
87	GLCM_Homogeneity	GLCM_Correlation	GLRLM_SRE	GLRLM_HGRE	NGLDM_Coarseness	GLZLM_ZLNU
88	GLCM_Correlation	GLRLM_HGRE	GLRLM_RP	NGLDM_Coarseness	GLZLM_SZLGE	GLZLM_SZHGE
89	GLCM_Homogeneity	GLRLM_RP	NGLDM_Coarseness	GLZLM_SZHGE	GLZLM_ZLNU	
90	GLCM_Homogeneity	GLRLM_HGRE	GLZLM_SZLGE	GLZLM_SZHGE	GLZLM_ZLNU	
91	GLCM_Homogeneity	GLCM_Correlation	GLRLM_HGRE	NGLDM_Coarseness	GLZLM_SZLGE	GLZLM_ZLNU
92	GLCM_Homogeneity	GLCM_Correlation	GLRLM_SRLGE	GLZLM_SZHGE		
93	GLCM_Homogeneity	NGLDM_Coarseness	GLZLM_SZLGE	GLZLM_SZHGE	GLZLM_ZLNU	
94	GLCM_Homogeneity	GLRLM_HGRE	NGLDM_Coarseness	GLZLM_SZLGE	GLZLM_SZHGE	GLZLM_ZLNU
95	GLCM_Homogeneity	GLCM_Correlation	GLRLM_SRLGE	NGLDM_Coarseness	GLZLM_SZLGE	GLZLM_SZHGE
	GLZLM_ZLNU					
96	GLCM_Homogeneity	GLCM_Correlation	GLRLM_HGRE	NGLDM_Coarseness	GLZLM_SZHGE	GLZLM_ZLNU
97	GLCM_Correlation	GLRLM_HGRE	GLRLM_RP	GLZLM_LGZE	GLZLM_SZLGE	GLZLM_ZLNU
98	GLRLM_HGRE	GLRLM_SRLGE	GLRLM_RP	NGLDM_Coarseness	GLZLM_ZLNU	
99	GLCM_Homogeneity	GLCM_Correlation	GLRLM_HGRE	GLRLM_SRLGE	GLZLM_SZHGE	GLZLM_ZLNU
100	GLCM_Homogeneity	GLRLM_SRE	GLRLM_HGRE	GLRLM_SRLGE	GLRLM_RP	NGLDM_Coarseness
	GLZLM_ZLNU					
