# Supplementary material for: Differentiation between Germinoma and Craniopharyngioma Using Radiomics-Based Machine Learning
Source: J Pers Med. 2022 Jan 4;12(1):45. doi: 10.3390/jpm12010045 (PMC8778008; doi:10.3390/jpm12010045)
Supplement: Supplementary file 1 [file jpm-12-00045-s001.zip › jpm-1511549-supplementary/Supplementary Material 2.pdf]

Contrast-enhanced T1WI Classifier: LDA	Distance Correlation	Random Forest	Lasso
Sensitivity_train	0.767857143	0.845201238	0.843
Specificity_train	0.901746725	0.861480076	0.92
Sensitivity_test	0.757894737	0.813333333	0.796
Specificity_test	0.824	0.772413793	0.843
AUC_train	0.909013613	0.927478054	0.966
AUC_test	0.888541339	0.858394547	0.914
accuracy_train	0.84	0.855294118	0.887
accuracy_test	0.795454545	0.786363636	0.823

Contrast-enhanced T1WI Classifier: SVM	Distance Correlation	Random Forest	Lasso
Sensitivity_train	0.579545455	1	0.693
Specificity_train	0.875776398	1	0.767
Sensitivity_test	0.587412587	error	0.654
Specificity_test	0.87012987	0.572727273	0.705
AUC_train	0.82887189	1	0.801
AUC_test	0.820774604	0.5	0.751
accuracy_train	0.691764706	1	0.739
accuracy_test	0.686363636	0.572727273	0.686

Contrast-enhanced T1WI<br>Classifier: RF	Distance Correlation	Random Forest	Lasso
Sensitivity_train	0.763157895	0.869918699	0.856
Specificity_train	0.832677165	0.948024948	0.864
Sensitivity_test	0.717647059	0.813186813	0.747
Specificity_test	0.755555556	0.84496124	0.738
AUC_train	0.89096405	0.971741597	0.949
AUC_test	0.785677236	0.910807917	0.832
accuracy_train	0.804705882	0.914117647	0.861
accuracy_test	0.740909091	0.831818182	0.741
