# Supplementary material for: Differentiation between Germinoma and Craniopharyngioma Using Radiomics-Based Machine Learning
Source: J Pers Med. 2022 Jan 4;12(1):45. doi: 10.3390/jpm12010045 (PMC8778008; doi:10.3390/jpm12010045)
Supplement: Supplementary file 1 [file jpm-12-00045-s001.zip › jpm-1511549-supplementary/Supplementary Material 3.pdf]

T2WI<br>Classifier: LDA	Distance Correlation	Random Forest	Lasso
Sensitivity_train	0.739263804	0.706051873	0.753
Specificity_train	0.837004405	0.838337182	0.897
Sensitivity_test	0.75	0.75	0.71
Specificity_test	0.810344828	0.851851852	0.822
AUC_train	0.884940576	0.852653033	0.92
AUC_test	0.880166764	0.850786783	0.875
accuracy_train	0.796153846	0.779487179	0.831
accuracy_test	0.785	0.805	0.77

T2WI<br>Classifier: SVM	Distance Correlation	Random Forest	Lasso
Sensitivity_train	0.603305785	1	0.592668024
Specificity_train	0.922297297	1	0.916955017
Sensitivity_test	0.62601626	error	0.636363636
Specificity_test	0.896103896	0.575	0.898734177
AUC_train	0.868855237	1	0.8572263
AUC_test	0.873151168	0.5	0.862297897
accuracy_train	0.724358974	1	0.712820513
accuracy_test	0.73	0.575	0.74

T2WI<br>Classifier: RF	Distance Correlation	Random Forest	Lasso
Sensitivity_train	0.798657718	0.829508197	0.846
Specificity_train	0.840248963	0.869473684	0.882
Sensitivity_test	0.69047619	0.674698795	0.682
Specificity_test	0.767241379	0.752136752	0.765
AUC_train	0.915251592	0.94806841	0.945
AUC_test	0.844366779	0.783381778	0.832
accuracy_train	0.824358974	0.853846154	0.868
accuracy_test	0.735	0.72	0.73
